# Prevalence, Awareness, Treatment, and Control of Hypertension in Korea

**DOI:** 10.1038/s41598-019-46965-4

**Published:** 2019-07-29

**Authors:** Si-Hyuck Kang, Sun-Hwa Kim, Jun Hwan Cho, Chang-Hwan Yoon, Seung-Sik Hwang, Hae-Young Lee, Tae-Jin Youn, In-Ho Chae, Cheol-Ho Kim

**Affiliations:** 1 Cardiovascular Center, Seoul National University Bundang Hospital, Seongnam-si, Korea; 2 Department of Internal Medicine, Seoul National University, Seoul, Korea; 3 Graduate School of Public Health, Seoul National University, Seoul, Korea; 4 Cardiovascular Center, Seoul National University Hospital, Seoul, Korea

**Keywords:** Risk factors, Cardiovascular biology

## Abstract

The purpose of the present study was to describe the temporal trends in prevalence and management status of hypertension in Korea between 1998 and 2015. Data of adults who were aged 30 years or older were extracted from the Korea National Health and Nutrition Examination Survey, a nationwide representative population-based survey. Hypertension was prevalent in 30.5% of Korean adults. The age and sex standardized prevalence showed little change between 1998 and 2015. The elderly population and men showed higher prevalence. The rates of awareness, treatment, and control showed substantial improvements among hypertensive subjects between 1998 and the time period of 2007‒2009 (awareness, from 23.5 to 66.3%; treatment, from 20.4 to 60.3%; and control, from 4.9 to 42.1%), after which the numbers reached a plateau and no significant changes were observed subsequently (67.3%, 63.6%, and 46.2%, respectively, between 2013 and 2015). The management status remained poor especially among the young population and in men. In conclusion, the hypertension prevalence remained stable at approximately 30% in Korea between 1998 and 2015. While awareness, treatment, and control of hypertension improved remarkably, the young population and particularly men showed a suboptimal management status.

## Introduction

Hypertension is a leading health risk^1^. The World Health Organization estimates that approximately 40% of adults have hypertension^2^. Annually, 9.4 million deaths are attributable to complications from elevated blood pressure (BP)^3,4^. Elevated BP accounts for 45% of all cardiovascular mortalities, and 51% of stroke-related deaths^2^.

Studies have reported that the global prevalence of hypertension has continuously risen during recent decades along with the trend of global aging^5^. Because hypertension is the most important contributing risk factor for disease burden, the importance of adequate prevention, diagnosis, and control of hypertension is emphasized more than ever before. Globally, however, less than half of all individuals with elevated BP are aware of their diagnosis, and less than one third of those under treatment show adequately controlled BP^6,7^.

The rates of awareness, treatment, and control of hypertension have shown to vary according to the region, sex, income, and educational levels^6,8–12^. While it is of great importance to recognize the current status in hypertension diagnosis and treatment at the country level, there are limited data published from Korea^13,14^. In Korea, a national surveillance program called the Korea National Health and Nutrition Examination Survey (KNHANES), is conducted annually since 1998 to assess the health and nutritional status at the national level^15^. To obtain nationally representative data, the survey includes approximately 10,000 individuals each year, and includes a health interview, health examination, and nutrition survey.

This study aimed to report the temporal trends in the prevalence, awareness, treatment, and control of hypertension in Korea using the nationwide survey data from 1998 to 2015. We analysed the epidemiology among various demographic and socioeconomic subgroups in an attempt to identify methods to improve hypertension management in Korea.

## Methods

### Ethics committee approval

This study was conducted in accordance with the Declaration of Helsinki, and the protocol was approved by the Seoul National University Hospital Institutional Review Board. Due to the retrospective nature of this study, the requirement of patient’s informed consent was waived.

### Data source

KNHANES is a nationwide representative survey that examines the general health and nutritional status of Koreans^15^. The first three surveys were conducted in 1998, 2001, and 2005. The design of KNHANES was modified from being conducted once every 3 years to every year beginning in 2007 (KNHANES IV includes data from 2007 to 2009, KNHANES V from 2010 to 2012, and KNHANES VI from 2013 to 2015). The number of subjects that were surveyed was approximately 35,000 for each survey during the first three surveys (1998, 2001 and 2005). Subsequently, in 2007, the survey included 5,000 subjects, which then increased to 10,000 per year since 2008. Study subjects were surveyed using a complex, stratified, multistage, cluster sampling. The sample weights are constructed to represent the Korean population by accounting for the complex survey design, survey non-response, and post-stratification. While the KNHANES includes subjects of all age groups (1 year and older), we included individuals who were 30 years or older in this study.

### Variables

A health interview, health examination, and nutrition survey are included in the KNHANES. Information on age, sex, income, and education level was obtained using standardized questionnaires during a home interview performed by trained medical personnel. In this study, income levels were categorized into quartiles according to the subject’s yearly household income. Educational status was categorized as no education or primary school graduate (primary), middle school graduate (middle), high school graduate (high), or college or university graduate or above (college/university). Area of residence was classified into urban or rural areas.

Health examination procedures were performed based on standardized protocols by trained medical personnel. All equipment was calibrated periodically. BP was measured three times on the subjects’ right arm using an appropriately sized arm cuff and mercury sphygmomanometer (Baumanometer; WA Baum Co., New York, NY, USA) after the subject rested in a seated position for at least 5 minutes. The final BP value was calculated as the average of the second and third measurements^16^. Hypertension was defined as systolic BP ≥ 140 mmHg, diastolic BP ≥ 90 mmHg, or use of antihypertensive drugs^17^. In this study, a subject was considered being aware of hypertension if he/she had a medical diagnosis of hypertension by medical personnel. Treatment rate was calculated by dividing the number of subjects taking antihypertensive medications for more than 20 days per month by the number of subjects with hypertension. Adequate BP control was defined as having an average systolic and diastolic BP of < 140/90 mmHg. Control rate (1) was defined as the proportion of subjects with adequate BP control among those with hypertension. Control rate (2) was defined as the proportion of subjects with adequate BP control among those who were receiving hypertension treatment.

### Statistical analysis

Data are presented as mean ± standard error or % (standard error). Sampling weights based on the sample design of each KNHANES were used for all statistical analyses^15^. Since a complex survey design was used in KNHANES, the rates of hypertension prevalence, awareness, treatment, and control were estimated by weighted means to avoid biased estimates. The variables representing strata, cluster, and weight were included in the raw data. The rates of awareness, treatment, and control of hypertension were then estimated by combining the 3 years from each survey (KNHANES IV, 2007–2009, KNHANES V, 2010–2012, and KNHANES VI, 2013–2015) using the integrated weights. Age standardization was done based on the 2005 Korean population to compare temporal trends, except for hypertension control rate (2) (adequate control of hypertension among subjects who were receiving antihypertensive medications) because the number of subjects in the age group of 30–39 years was less than 20. Statistical analyses were conducted using SPSS Statistics (IBM Corp., Armonk, NY, USA) and R programming version 3.2.4 (http://www.R-project.org; The R Foundation for Statistical Computing, Vienna, Austria).

## Results

### Characteristics of hypertensive subjects

Table 1 shows the profile of subjects with hypertension during the study period. The mean age of hypertensive subjects was 59.8 years in 2013‒2015, which showed a gradual increase from 54.9 years in 1998. Among those with hypertension, 53.1% were men, 21.2% had diabetes; 60.4% dyslipidaemia; and 50.1% were obese. The mean total cholesterol, and LDL cholesterol were 191.2 ± 0.7 and 112.4 ± 0.8, respectively.

**Table 1:** Characteristics of subjects with hypertension.

	KNHANES I (1998)	KNHANES II (2001)	KNHANES III (2005)	KNHANES IV (2007–2009)	KNHANES V (2010–2012)	KNHANES VI (2013–2015)
Number of subjects	(n = 6,435)	(n = 5,029)	(n = 4,795)	(n = 14,935)	(n = 15,738)	(n = 13,657)
Age	54.9 ± 0.4	57.7 ± 0.5	57.1 ± 0.5	58.5 ± 0.3	59.2 ± 0.3	59.8 ± 0.3
**Sex**						
Male	51.4%	47.8%	53.8%	52.1%	51.8%	53.1%
Female	48.6%	52.2%	46.2%	47.9%	48.2%	46.9%
**Anthropometric measurements**						
Body mass index (kg/m^2^)	24.4 ± 0.1	24.8 ± 1.1	25.1 ± 1.1	25.0 ± 0.1	25.0 ± 0.1	25.2 ± 0.1
Waist circumference (cm)	85.2 ± 0.3	86.2 ± 0.3	86.7 ± 0.3	86.3 ± 0.3	85.9 ± 0.2	86.4 ± 0.2
Systolic blood pressure (mmHg)	150.7 ± 0.6	146.2 ± 0.6	138.8 ± 0.7	134.9 ± 0.4	135.4 ± 0.3	133.4 ± 0.3
Diastolic blood pressure (mmHg)	91.6 ± 0.3	89.0 ± 0.4	88.1 ± 0.6	84.9 ± 0.3	83.1 ± 0.2	81.5 ± 0.3
Heart rate (/min)	73.5 ± 0.3	73.4 ± 0.4	68.3 ± 0.4	65.0 ± 1.7	60.5 ± 0.9	58.5 ± 0.7
**Lifestyle behaviours**						
Regular physical activity	—	—	—	47.4%	35.5%	36.3%
Heavy alcohol drinking	—	—	—	15.2%	14.3%	14.2%
Daily calorie intake (kcal)	1871.7 ± 26.2	1846.5 ± 29.0	1954.0 ± 32.9	1795.2 ± 19.2	1923.5 ± 18.6	1948.9 ± 18.6
Daily sodium consumption (mg)	4865.7 ± 111.8	5428.4 ± 113.7	5551.8 ± 112.7	4681.8 ± 68.5	4766.6 ± 64.9	3742.9 ± 50.7
**Comorbidities**						
Obesity	40.9 ± 1.4	46.9 ± 1.5	50.6 ± 1.5	49.1 ± 0.9	47.3 ± 0.9	50.1 ± 0.9
Diabetes mellitus	—	—	16.2 ± 1.2	20.0 ± 0.7	19.5 ± 0.7	21.2 ± 0.8
Dyslipidaemia	—	—	60.6 ± 1.6	59.8 ± 1.0	57.6 ± 0.9	60.4 ± 0.9
**Laboratory findings**						
Total cholesterol (mg/dL)	200.8 ± 1.0	199.8 ± 1.1	194.0 ± 1.2	195.5 ± 0.8	193.7 ± 0.7	191.2 ± 0.7
HDL cholesterol (mg/dL)	48.5 ± 0.3	44.5 ± 0.4	43.0 ± 0.3	45.9 ± 0.2	47.4 ± 0.2	48.5 ± 0.2
LDL cholesterol (mg/dL)	124.1 ± 1.1	122.7 ± 1.1	119.0 ± 1.0	115.3 ± 1.9	116.7 ± 1.2	112.4 ± 0.8
Triglyceride (mg/dL)	145.4 ± 1.7	165.4 ± 2.6	185.3 ± 6.1	173.2 ± 2.8	166.1 ± 2.2	169.7 ± 2.6
Fasting glucose (mg/dL)	109.4 ± 1.1	102.4 ± 0.6	103.5 ± 1.0	106.3 ± 0.5	105.1 ± 0.5	107.7 ± 0.5
Calculated GFR (mL/min/1.73 m^2^)	51.2 ± 0.5	57.9 ± 0.6	44.7 ± 0.3	53.7 ± 0.5	51.1 ± 0.3	53.1 ± 0.7

Data are presented as mean ± SE or % (SE). Log transformed geometric means are presented for triglycerides. HDL denotes high density lipoprotein; LDL, low density lipoprotein; GFR, glomerular filtration rate.

### Prevalence of hypertension

**Figure 1:**
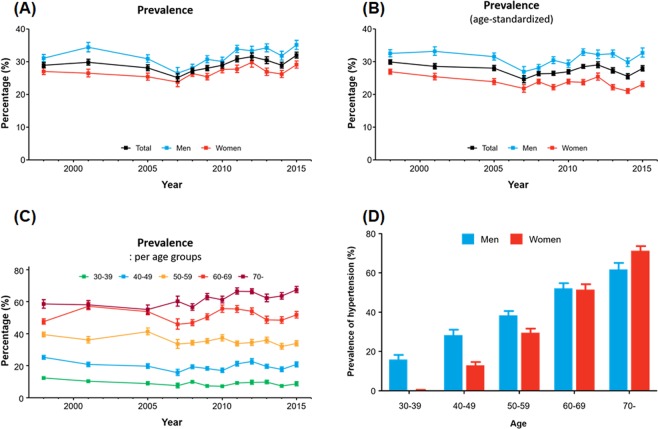
Trends in the prevalence of hypertension in Korea: (**A**) crude prevalence, (**B**) age-standardized prevalence, (**C**) prevalence across different age groups, and (**D**) prevalence according to sex and age in 2013‒2015.

Prevalence of hypertension was 28.9% in 1998, lowest in 2007 (25.1%), and slightly higher in 2015 (32.0%) (Fig. 1A). This fluctuation seemed to be due to the aging population (Supplementary Fig. 1). When standardized with age distribution, the prevalence of hypertension was grossly stable (Fig. 1B). The prevalence of hypertension in each age group remained unchanged during the study period (Fig. 1C). Hypertension was more prevalent in men than in women (35.1% and 29.1%, respectively, in 2015) (Table 2). While both sexes showed increasingly higher prevalence with older ages, men and women exhibited different patterns (Fig. 1D). For example, among those who were between 30‒39 years, the proportion of hypertensive individuals was markedly higher among men (15.9%) than among women (1.6%). However, the overall rate of hypertension was similar in men and women aged 60‒69 years, and eventually became higher among women than men among those aged 70 years or older. The prevalence did not differ to a large degree according to the area of residence (Supplementary Fig. 2A); however, prevalence tended to be higher in lower income quartiles and lower education levels (Supplementary Fig. 2B,C). Detailed data on the prevalence of hypertension are described in Supplementary Tables 1‒3, which present the prevalence of hypertension from 1998 to 2015 in all subjects and by sex.

**Table 2:** Prevalence, Awareness, Treatment and Control of Hypertension in Korea (2013–2015).

	Prevalence	Awareness	Treatment	Control (1)	Control (2)
Total	30.5 ± 0.6	67.3 ± 0.9	63.6 ± 0.9	46.2 ± 1.0	72.0 ± 1.0
**Sex**					
Male	33.7 ± 0.8	60.1 ± 1.4	55.4 ± 1.4	40.4 ± 1.3	72.4 ± 1.4
Female	27.4 ± 0.6	75.4 ± 1.0	72.7 ± 1.0	52.7 ± 1.2	71.7 ± 1.3
**Age (years)**					
30–39	8.6 ± 0.6	20.2 ± 3.2	15.2 ± 2.8	9.8 ± 2.2	64.7 ± 9.3
40–49	19.4 ± 0.9	43.9 ± 2.5	39.5 ± 2.6	29.8 ± 2.4	74.9 ± 3.5
50–59	34.0 ± 1.0	61.4 ± 1.8	56.2 ± 1.8	40.1 ± 1.8	70.7 ± 2.2
60–69	49.7 ± 1.2	82.0 ± 1.2	79.3 ± 1.3	59.6 ± 1.6	74.5 ± 1.6
70+	64.5 ± 1.2	86.0 ± 0.9	83.4 ± 1.0	59.3 ± 1.4	70.4 ± 1.5
**Area of residence**					
Urban area	29.5 ± 0.6	66.9 ± 1.1	62.8 ± 1.1	45.9 ± 1.1	72.4 ± 1.1
Rural area	34.5 ± 1.4	68.8 ± 1.9	66.3 ± 2.0	47.3 ± 2.2	70.9 ± 2.1
**Income quartiles**					
Highest	28.7 ± 1.0	68.0 ± 1.6	63.6 ± 1.7	46.9 ± 1.7	73.1 ± 1.8
Upper middle	29.5 ± 0.9	65.9 ± 1.9	62.8 ± 1.8	43.9 ± 1.8	69.2 ± 2.1
Lower middle	31.3 ± 1.0	63.8 ± 1.7	60.4 ± 1.8	45.9 ± 1.8	75.1 ± 1.8
Lowest	32.4 ± 1.0	71.3 ± 1.7	67.6 ± 1.8	48.2 ± 1.9	71.0 ± 1.9
**Education levels**					
Primary	80.2 ± 1.0	80.2 ± 1.0	77.3 ± 1.1	54.9 ± 1.4	70.4 ± 1.4
Middle	69.2 ± 2.1	69.2 ± 2.1	63.8 ± 2.2	47.7 ± 2.3	74.1 ± 2.5
High	62.0 ± 1.8	62.0 ± 1.8	47.0 ± 2.8	44.6 ± 1.8	75.9 ± 1.7
University/college	48.5 ± 2.3	48.5 ± 2.3	44.4 ± 2.2	31.6 ± 2.0	70.9 ± 2.7

Data are presented as mean ± SE. Control rate (1), the rate of adequate blood pressure control among individuals with hypertension. Control rate (2), the rate of adequate blood pressure control among those under hypertension treatment.

### Awareness of hypertension

**Figure 2:**
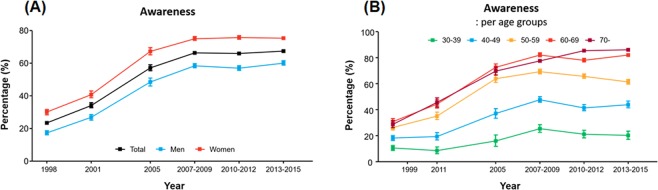
Trends in the awareness of hypertension in Korea: (**A**) overall awareness and (**B**) awareness across different age groups.

Awareness of hypertension dramatically improved over the decades—from 23.5% in 1988 to 67.3% in 2013‒2015 (Fig. 2A) (Supplementary Fig. 3A, which shows age-standardized rates). However, the rate reached a plateau in the period of 2007‒2009 and did not significantly change thereafter. Women were more frequently aware of having hypertension than men. When stratified into age groups, the awareness rate showed a wide variation: >80% among those aged 60 years or older, 61.4% among those aged 50‒59 years, 43.9% among those aged 40‒49 years, and 20.2% among those aged 30‒39 years (Fig. 2B). This pattern was similar between men and women (Supplementary Tables 4‒6). The rate of awareness did not differ significantly between urban and rural areas, or among income quartiles (Supplementary Fig. 3B,C); no consistent gradient according to the education levels was observed (Supplementary Fig. 3D).

### Treatment of hypertension

**Figure 3:**
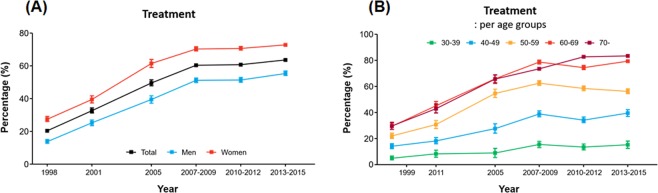
Trends in the treatment of hypertension in Korea: (**A**) overall treatment and (**B**) treatment across different age groups.

The rate of hypertension treatment had a similar pattern as the awareness rate. While 20.4% of subjects with hypertension received treatment in 2006, the number went up to 63.6% in 2013‒2015 (Fig. 3A). Treatment rate plateaued in the period of 2007‒2009, with no significant improvement thereafter. The pattern of the rate of treatment in each age group was similar to that observed for awareness rate (Fig. 3B). There were small gaps between the awareness and treatment rates, although these gaps tended to be greater in younger age groups (Supplementary Tables 7‒9). No significant variations were found according to the residence, income, and education (Supplementary Fig. 4).

### Control of hypertension

**Figure 4:**
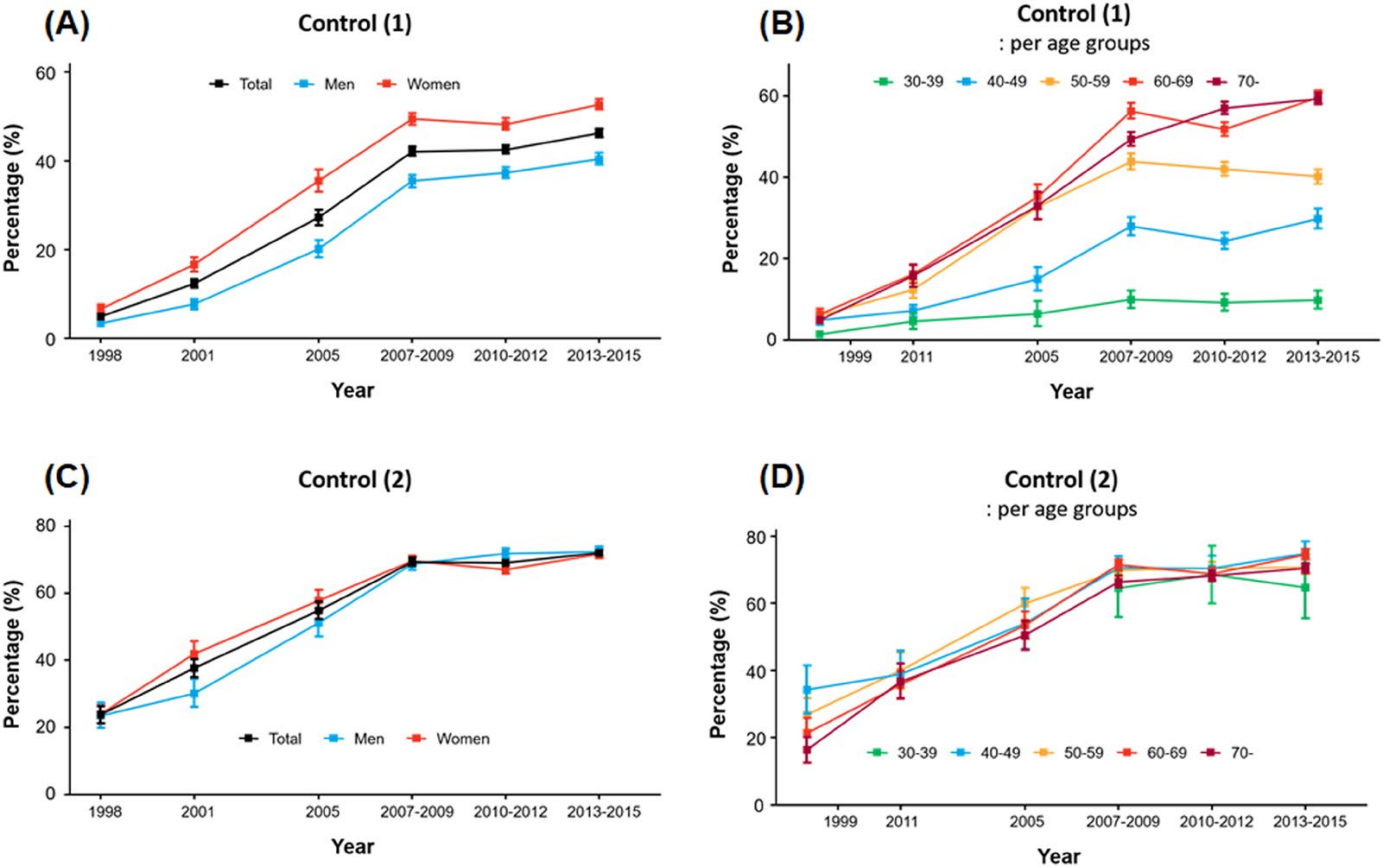
Trends in the control of hypertension in Korea. Control (1) is the rate of control among subjects with hypertension: (**A**) overall control and (**B)** control across different age groups. Control (2) is the rate of control among subjects with hypertension who were receiving treatment: (**C**) overall control and (**D**) control across different age groups.

Among hypertensive patients, adequately controlled BP (control rate [1]) markedly improved from 1998 (4.9%) to the period of 2013‒2015 (46.2%) (Fig. 4A). The rate greatly varied according to the subjects’ age — approximately 60% among elderly compared to <10% among 30‒39-year-olds (see Fig. 4B) (Supplementary Tables 10‒12).

Among hypertensive patients who were receiving treatment (control rate [2]), 72.0% had an adequate level of BP control compared to 23.8% in 1998 (Fig. 4C). However, different from the prevalence, awareness, treatment, and control (1) rates, as described above, control rate (2) showed little variation among different age groups and between sexes (Fig. 4D) (Supplementary Tables 13‒15). The subjects’ other socioeconomic factors did not significantly affect the control rate (Supplementary Figs 5 and 6).

**Figure 5:**
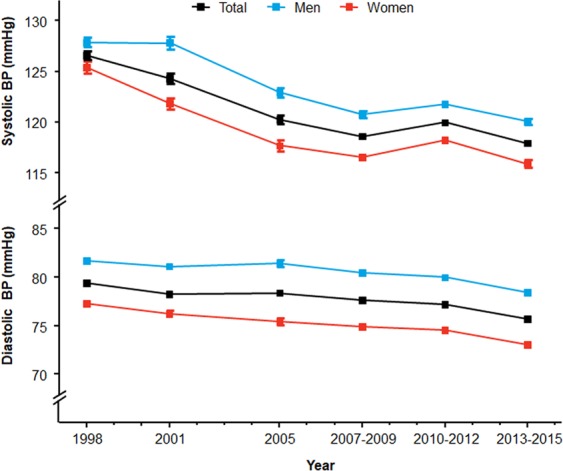
Temporal trends of mean blood pressure in the general Korean population.

Overall, the improvement in detection and management of hypertension contributed to observed decrease in BP in the general Korean population: 127/79 mmHg in 1998, to 118/76 mmHg in 2013‒2015 (Fig. 5). This trend was also consistent between sexes and among the age groups (Supplementary Fig. 7). The absolute degree of reduction was greater among the aged population and in women.

## Discussion

In this study, we found that as of 2015, 30.5% of Koreans had hypertension. Among hypertensive subjects, 67.3% were aware of their diagnosis, 63.6% received treatment, and 46.2% had adequately controlled BP. The awareness, treatment, and control rates significantly improved since 1998, when the first KNHANES survey was conducted. However, they reached a plateau in 2007‒2009, and no significant changes were detected thereafter. We also found that there is still room for improvement among people with hypertension who are young and in hypertensive men, as they showed the lowest levels of awareness, treatment, and control.

This study showed that the prevalence of hypertension in Korea remained grossly unchanged from 1998 to 2015. The crude prevalence showed modest fluctuations, but the age-adjusted prevalence was stationary during the study period. Male sex and old age were associated with higher prevalence of hypertension, while there were little disparities according to area of residence, income level, and education. Previous studies have reported wide variations in the prevalence of hypertension across geographic regions (Supplementary Table 16)^6,8–12^. The prevalence in Korea shown in this study was among the lowest and was similar to that in the United States^8,9^. The prevalence was lower than the rate reported in Japan, which shares a similar ethnic background, geography, and lifestyle as Korea^10,18^. However, when split into age and sex subgroups, both Korea and Japan displayed a similar prevalence of hypertension. As the Korean population is rapidly aging, the prevalence of hypertension is expected to constantly increase in the future.

There was a dramatic improvement in the identification and control of hypertension during the study period. The awareness and treatment rates increased more than three-fold, and the control rate increased approximately ten-fold between 1998 and the period of 2013‒2015. The overall control status observed in Korea was among the best globally (Supplementary Table 16). Several changes in the Korean society may have influenced this phenomenon. First, daily sodium consumption has decreased; the mean sodium intake is relatively high in Korea compared to global levels^19^. However, the amount was reported to be decreasing among the Korean population^20^, and this study also confirmed that sodium consumption was decreasing among those with hypertension. Second, there has been an increase in nationwide efforts towards early detection of hypertension. For example, subscribers of the National Health Insurance system, which covers 97% of the Korean population, are entitled to receive a standardized medical examination every 2 years since 2011^21^. This free service includes general physical examination and laboratory tests, such as fasting glucose and blood lipid levels. If a recipient’s BP is high, he/she is recommended to visit a primary physician for further evaluation. In 2015, over 60% of Korean adults received the free screening service. In addition, the government, in conjunction with professional societies, is implementing various measures for the primary prevention of chronic diseases. Third, there have been advances in healthcare policy. For example, a pharmaceutical policy reform which was enforced in 2000, mandated the separation of drug prescription and dispensing^22^. While the policy aimed at reducing the overuse and misuse of drugs, it inevitably increased medical service consumers’ visits to physicians. These changes in practice patterns may have contributed to the improvement in hypertension awareness and treatment.

Recent data demonstrated a significant decrease in mortality from cardiovascular diseases in Korea^23^. A more dramatic reduction was observed in cerebrovascular disease incidence and mortality^24^. This study indicated that the mean BP of the general Korean population declined by 8.6/3.7 mmHg over the last 18 years. There is strong evidence suggesting that a BP reduction of 10/5 mmHg is associated with an 18% reduction in cardiovascular mortality^25,26^. Therefore, it may be assumed that the improvement in hypertension management was one of the major contributors to the recent reductions in cardiovascular mortality.

This study revealed low rates of detection and poor control of hypertension among Korean individuals who are young and in men, which is a common observation globally^6,8–12^, though the gaps are smaller in several countries such as the United States, Japan, and Germany^8,18,27^. Of note, the countries listed above also showed better awareness and management of hypertension than in Korea, which provides an important insight. Early detection should be encouraged, and antihypertensive treatment should be commenced as soon as clinically indicated for young Korean men. These early interventions might also assist in breaking through the stagnation of awareness, treatment, and control rates that have occurred since 2007‒2009.

Measures to improve hypertension management in individuals who are young and male are required. Previous studies have shown that infrequent healthcare visits are an important risk factor for low awareness and management^28^. A study in Korea also showed that only a small proportion of young patients (in their 30 s and 40 s) with hypertension received BP measurements during previous years^29^. Self-measured BP at home has shown to be an effective measure to improve adherence to treatment^30^. In addition, there are other methods that have shown efficacy, such as simplification of the drug regimen and home-based digital programs^31,32^.

American College of Cardiology (ACC) and American Heart Association (AHA) recently published a new guideline for hypertension, which suggested more aggressive diagnostic and treatment approaches than before^33^. According to these, hypertension was defined as a BP ≥ 130/80 mmHg and hypertensive patients were recommended to maintain their BP under <130/80 mmHg. However, European hypertension guidelines as well as Korean ones have maintained the previous cut-off value of 140/90 mmHg^34^. In a previous study, we demonstrated that the prevalence of hypertension would increase from 30.4% to 49.2% when the 2017 ACC/AHA guidelines are directly applied in Korea^35^.

The major strength of this study was the use of the nationally representative KNHANES data. It enabled reliable estimation of the trends of hypertension in Korea over 18 years. The limitations of this study also need to be considered. First, the KNHANES is designed as a cross-sectional study and longitudinal follow-up is limited^36^. Second, there have been modifications in the questionnaires from KNHANES I to VI; thus, there were minor variations in the definitions of hypertension across the study period. Third, BP measurements were performed during a single visit. Repeated visits or ambulatory measurements have shown to minimize the chance of misclassification^37^. Lastly, the Korean Society of Hypertension recently published the Korea Hypertension Fact Sheet 2018 in Korean^38^. Some of the present study’s results are similar to those of the Fact Sheet.

In conclusion, this study reported the trends in hypertension prevalence, awareness, treatment, and control in Korea. Among Korean adults aged 30 years or older, 30.4% had hypertension. While the prevalence was largely unchanged, the awareness, treatment, and control rates drastically improved from 1998 to 2007, at which time the rates plateaued and remained constant subsequently. Strategies need to be implemented to increase hypertension detection, management, and control among young people and men in Korea.

## Supplementary information

Supplementary appendix
