# Supplementary material for: Prevalence, Awareness, Treatment, and Control of Hypertension in Korea
Source: Sci Rep. 2019 Jul 29;9:10970. doi: 10.1038/s41598-019-46965-4 (PMC6662850; doi:10.1038/s41598-019-46965-4)

**Supplementary Appendix**

**Prevalence, Awareness, Treatment, and Control of Hypertension in Korea**

Si-Hyuck Kang^a,b^, Sun-Hwa Kim^a^, Jun Hwan Cho^a,b^, Chang-Hwan Yoon^a,b^, Seung-Sik Hwang^c^,

Hae-Young Lee^b,d^, Tae-Jin Youn^a,b^, In-Ho Chae^a,b^, Cheol-Ho Kim^a,b,*^

^a^Cardiovascular Center, Seoul National University Bundang Hospital, Seongnam-si, Korea; ^b^Department of Internal Medicine, Seoul National University, Seoul, Korea; ^c^Graduate School of Public Health, Seoul National University, Seoul, Korea; ^d^Cardiovascular Center, Seoul National University Hospital, Seoul, Korea

**Supplementary Figure 1.** Changes in population distribution in Korea.


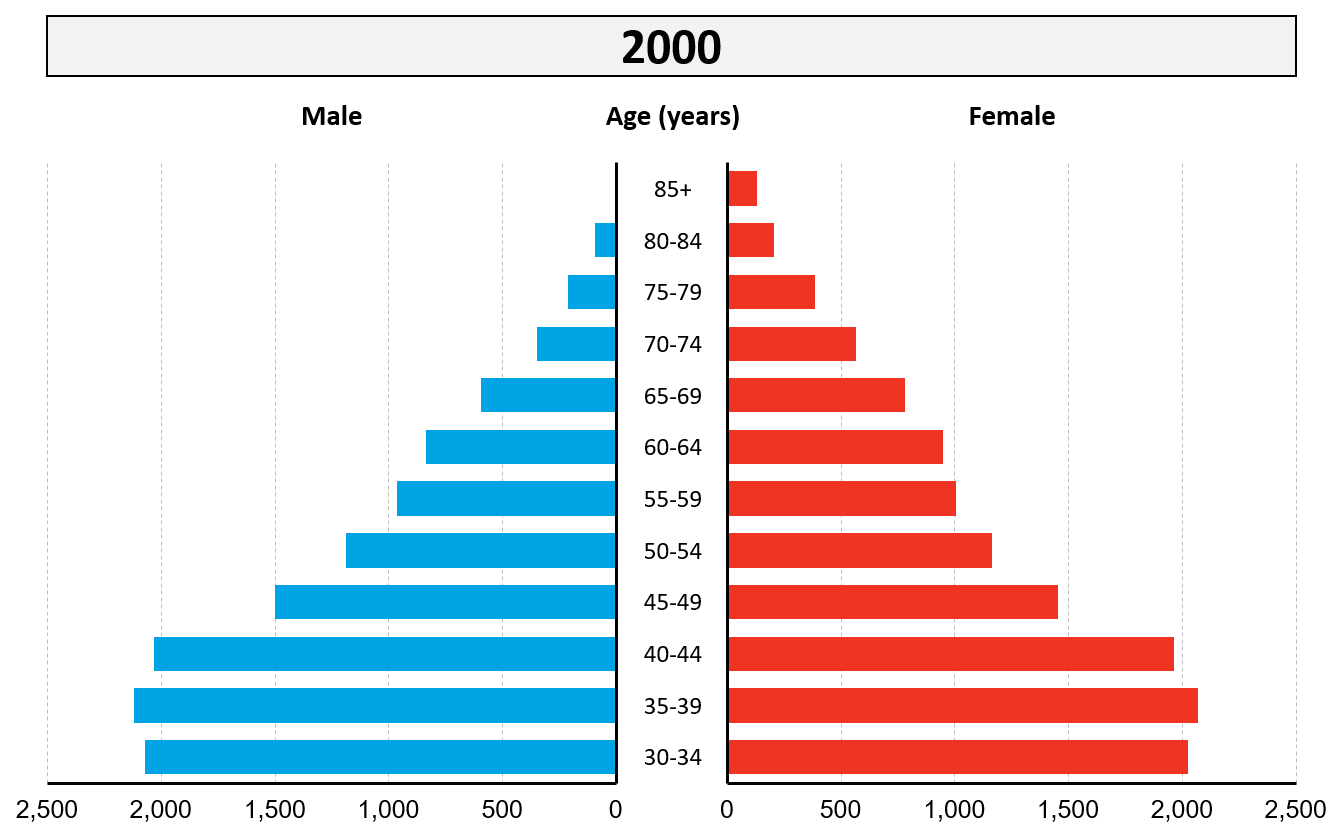

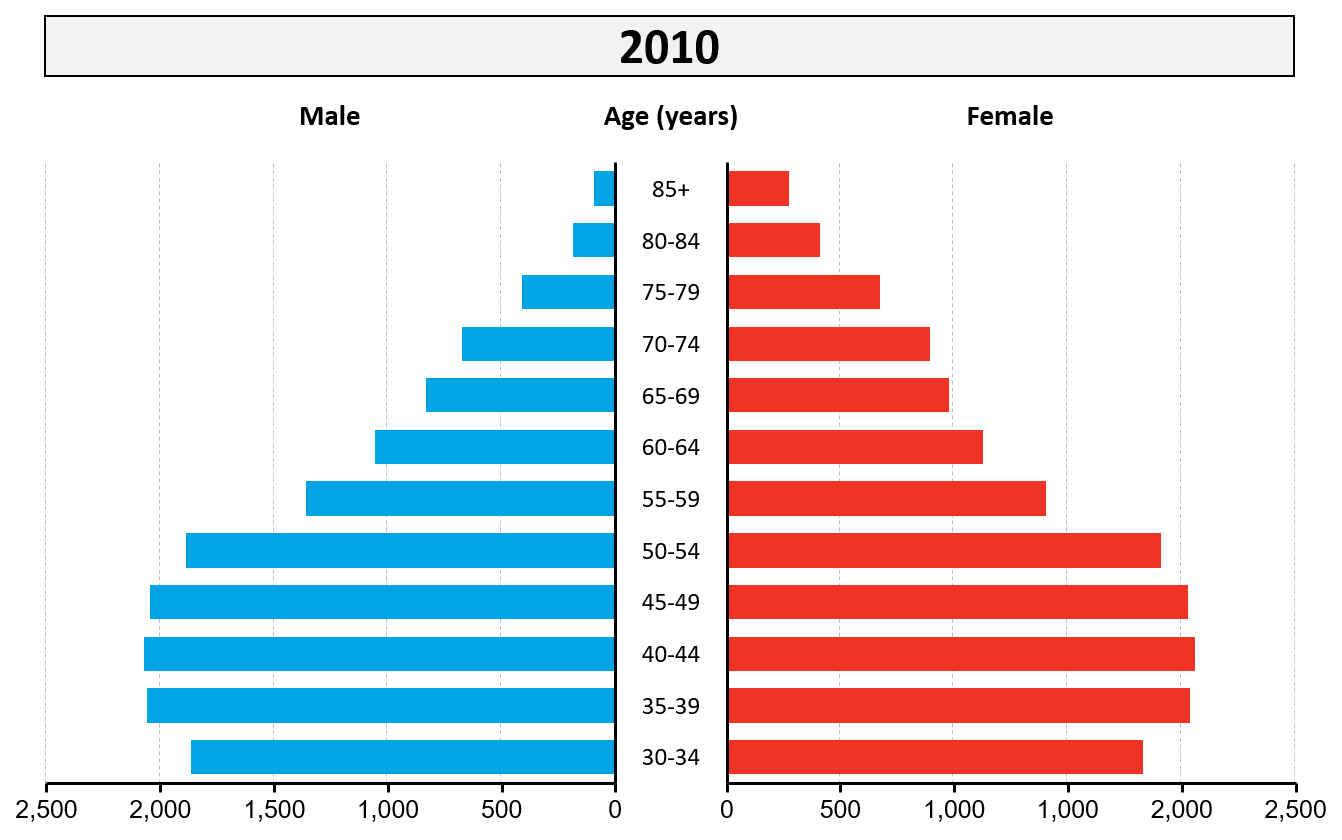


Data were obtained from Korea population census (KOrean Statistical Information Service, http://kosis.kr/eng/; accessed in Oct 2017)

**Supplementary Figure 2.** Age-standardized prevalence of hypertension according to (A) the area of residence, (B) income quartiles, and (C) education levels

| **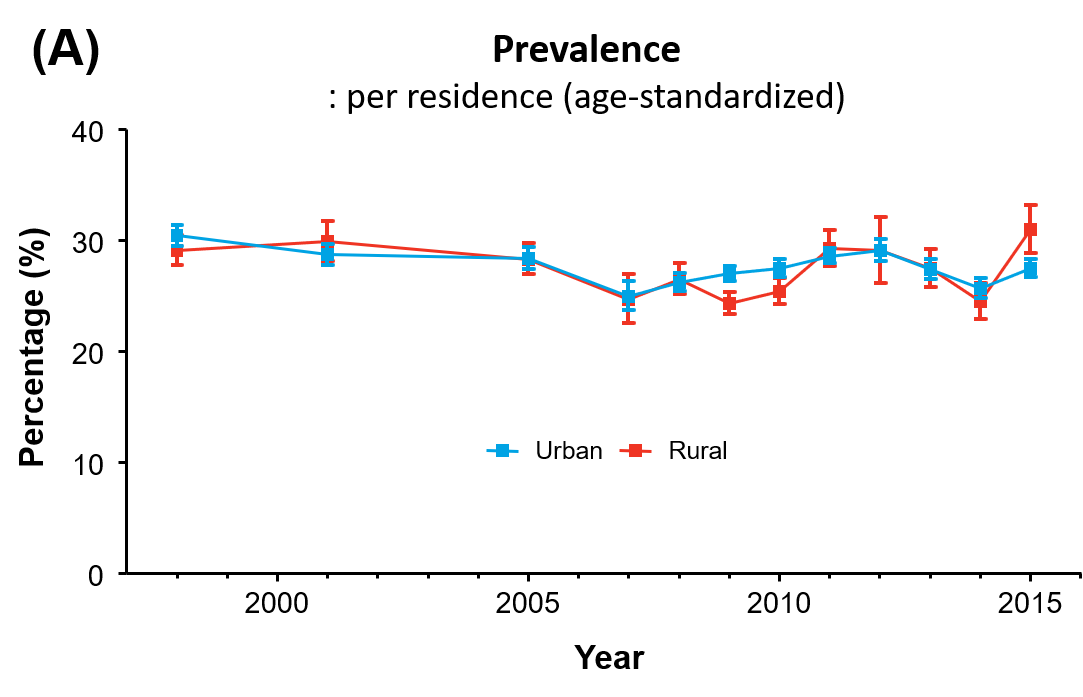** | **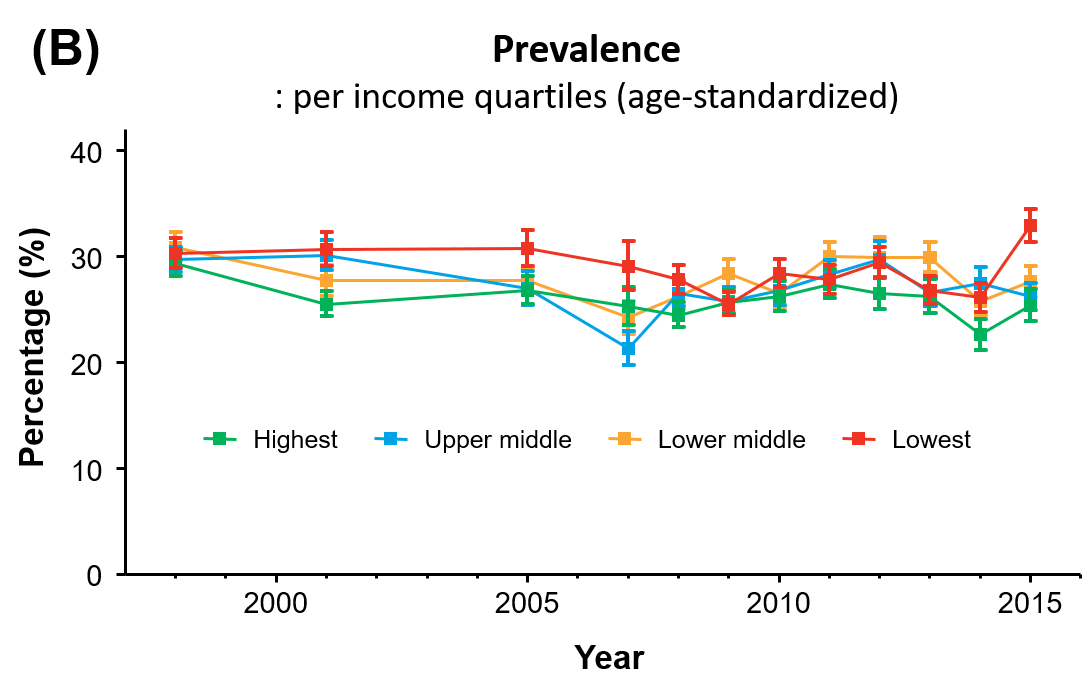** |
| --- | --- |
| **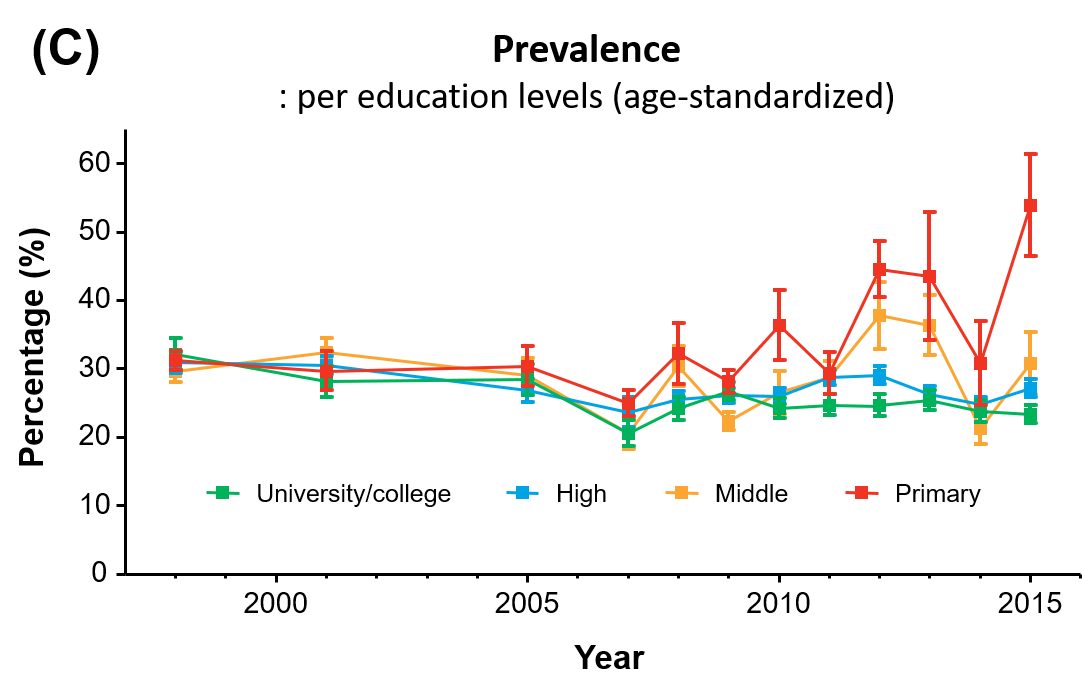** |  |

**Supplementary Table 1.** Prevalence of hypertension, 1998‒2015

|  | 1998 | 2001 | 2005 | 2007 | 2008 | 2009 | 2010 | 2011 | 2012 | 2013 | 2014 | 2015 |
| --- | --- | --- | --- | --- | --- | --- | --- | --- | --- | --- | --- | --- |
| Prevalence | 28.9 ± 0.8 | 29.8 ± 0.9 | 28.1 ± 0.9 | 25.1 ± 1.2 | 27.2 ± 0.8 | 28.0 ± 0.8 | 28.9 ± 0.9 | 30.8 ± 0.9 | 31.5 ± 1.1 | 30.4 ± 1.0 | 28.9 ± 0.9 | 32.0 ± 0.9 |
| Prevalence (standardized) | 29.9 ± 0.7 | 28.6 ± 0.8 | 28 ± 0.8 | 24.6 ± 1.1 | 26.3 ± 0.6 | 26.4 ± 0.6 | 26.9 ± 0.7 | 28.5 ± 0.6 | 29.0 ± 0.9 | 27.3 ± 0.8 | 25.5 ± 0.8 | 27.9 ± 0.8 |
| Age (years) |  |  |  |  |  |  |  |  |  |  |  |  |
| 30-39 | 12.3 ± 0.8 | 10.3 ± 0.9 | 8.8 ± 1.1 | 7.5 ± 1.4 | 9.9 ± 0.9 | 7.3 ± 0.9 | 7.2 ± 0.9 | 9.1 ± 1.0 | 9.5 ± 1.3 | 9.7 ± 1.1 | 7.3 ± 0.9 | 8.7 ± 1.3 |
| 40-49 | 25.2 ± 1.2 | 20.7 ± 1.3 | 19.7 ± 1.5 | 15.7 ± 1.9 | 19.3 ± 1.2 | 18.2 ± 1.1 | 17.0 ± 1.3 | 21.1 ± 1.5 | 22.6 ± 1.7 | 19.5 ± 1.3 | 17.7 ± 1.5 | 20.7 ± 1.6 |
| 50-59 | 39.5 ± 1.5 | 36.1 ± 2.0 | 41.3 ± 2.1 | 33.6 ± 2.8 | 34.2 ± 1.5 | 35.3 ± 1.5 | 37.3 ± 1.9 | 33.8 ± 1.6 | 34.5 ± 1.9 | 35.9 ± 1.8 | 32.0 ± 1.8 | 33.9 ± 1.6 |
| 60-69 | 47.5 ± 1.7 | 57.2 ± 2.3 | 53.7 ± 2.0 | 45.9 ± 3.2 | 46.8 ± 1.8 | 50.5 ± 1.8 | 55.6 ± 2.3 | 55.4 ± 2.1 | 54.0 ± 2.0 | 48.7 ± 2.2 | 48.5 ± 2.0 | 51.8 ± 2.0 |
| 70+ | 58.5 ± 2.6 | 58.1 ± 2.6 | 55.0 ± 2.9 | 60.2 ± 3.1 | 56.6 ± 2.0 | 63.1 ± 1.9 | 61.2 ± 2.1 | 66.6 ± 2.0 | 66.4 ± 1.6 | 62.3 ± 2.4 | 63.5 ± 2.0 | 67.5 ± 1.9 |
| Area of residence^*^ |  |  |  |  |  |  |  |  |  |  |  |  |
| Urban area | 30.4 ± 0.9 | 28.7 ± 0.9 | 28.4 ± 1.0 | 25.0 ± 1.3 | 26.2 ± 0.8 | 27.0 ± 0.7 | 27.5 ± 0.8 | 28.6 ± 0.7 | 29.1 ± 1.0 | 27.4 ± 0.9 | 25.7 ± 0.9 | 27.5 ± 0.8 |
| Rural area | 29.1 ± 1.3 | 29.9 ± 1.8 | 28.3 ± 1.4 | 24.7 ± 2.2 | 26.5 ± 1.4 | 24.3 ± 1.0 | 25.4 ± 1.2 | 29.3 ± 1.6 | 29.1 ± 3.0 | 27.5 ± 1.7 | 24.5 ± 1.6 | 31.0 ± 2.2 |
| Income quartiles^*^ |  |  |  |  |  |  |  |  |  |  |  |  |
| Highest | 32.1 ± 2.3 | 28.1 ± 2.2 | 28.4 ± 2.3 | 20.5 ± 1.9 | 24.2 ± 1.7 | 26.7 ± 1.3 | 24.2 ± 1.5 | 24.7 ± 1.5 | 24.6 ± 1.6 | 25.4 ± 1.5 | 23.8 ± 1.6 | 23.3 ± 1.3 |
| Upper middle | 30.9 ± 1.6 | 30.5 ± 1.3 | 26.8 ± 1.6 | 23.7 ± 2.2 | 25.5 ± 1.2 | 26.1 ± 1.1 | 26.0 ± 1.2 | 28.7 ± 1.1 | 29.1 ± 1.3 | 26.2 ± 1.3 | 24.8 ± 1.1 | 27.1 ± 1.3 |
| Lower middle | 29.7 ± 1.7 | 32.4 ± 2.0 | 29.0 ± 2.6 | 20.5 ± 2.3 | 30.4 ± 2.9 | 22.3 ± 1.3 | 26.5 ± 3.2 | 28.7 ± 2.4 | 37.8 ± 4.9 | 36.4 ± 4.4 | 21.3 ± 2.3 | 30.8 ± 4.6 |
| Lowest | 31.2 ± 1.4 | 29.7 ± 2.9 | 30.4 ± 2.9 | 24.9 ± 1.9 | 32.2 ± 4.4 | 28.1 ± 1.7 | 36.4 ± 5.1 | 29.3 ± 3.1 | 44.6 ± 4.1 | 43.5 ± 9.3 | 30.8 ± 6.1 | 53.9 ± 7.4 |
| Education levels^*^ |  |  |  |  |  |  |  |  |  |  |  |  |
| Primary | 31.2 ± 1.4 | 29.7 ± 2.9 | 30.4 ± 2.9 | 24.9 ± 1.9 | 32.2 ± 4.4 | 28.1 ± 1.7 | 36.4 ± 5.1 | 29.3 ± 3.1 | 44.6 ± 4.1 | 43.5 ± 9.3 | 30.8 ± 6.1 | 53.9 ± 7.4 |
| Middle | 29.7 ± 1.7 | 32.4 ± 2.0 | 29.0 ± 2.6 | 20.5 ± 2.3 | 30.4 ± 2.9 | 22.3 ± 1.3 | 26.5 ± 3.2 | 28.7 ± 2.4 | 37.8 ± 4.9 | 36.4 ± 4.4 | 21.3 ± 2.3 | 30.8 ± 4.6 |
| High | 30.9 ± 1.6 | 30.5 ± 1.3 | 26.8 ± 1.6 | 23.7 ± 2.2 | 25.5 ± 1.2 | 26.1 ± 1.1 | 26.0 ± 1.2 | 28.7 ± 1.1 | 29.1 ± 1.3 | 26.2 ± 1.3 | 24.8 ± 1.1 | 27.1 ± 1.3 |
| University/college | 32.1 ± 2.3 | 28.1 ± 2.2 | 28.4 ± 2.3 | 20.5 ± 1.9 | 24.2 ± 1.7 | 26.7 ± 1.3 | 24.2 ± 1.5 | 24.7 ± 1.5 | 24.6 ± 1.6 | 25.4 ± 1.5 | 23.8 ± 1.6 | 23.3 ± 1.3 |

Data are presented as mean ± SE. *age-standardized rate (standardized to the population in 2005).

**Supplementary Table 2.** Prevalence of hypertension in men, 1998‒2015

|  | 1998 | 2001 | 2005 | 2007 | 2008 | 2009 | 2010 | 2011 | 2012 | 2013 | 2014 | 2015 |
| --- | --- | --- | --- | --- | --- | --- | --- | --- | --- | --- | --- | --- |
| Prevalence | 31.1 ± 1.1 | 34.4 ± 1.4 | 30.9 ± 1.2 | 26.5 ± 1.7 | 28.1 ± 1.1 | 30.7 ± 1.1 | 30.1 ± 1.2 | 33.9 ± 1.1 | 33.3 ± 1.4 | 34.2 ± 1.2 | 31.8 ± 1.3 | 35.1 ± 1.4 |
| Prevalence (standardized) | 32.5 ± 1.1 | 33.2 ± 1.3 | 31.5 ± 1.1 | 26.9 ± 1.6 | 28.1 ± 1.0 | 30.4 ± 1.0 | 29.3 ± 1.1 | 32.9 ± 1.0 | 32.2 ± 1.3 | 32.4 ± 1.1 | 29.8 ± 1.3 | 32.7 ± 1.4 |
| Age (years) |  |  |  |  |  |  |  |  |  |  |  |  |
| 30-39 | 18.6 ± 1.4 | 17.6 ± 1.6 | 14.1 ± 1.8 | 13.3 ± 2.6 | 15.2 ± 1.6 | 11.5 ± 1.5 | 12.6 ± 1.6 | 14.6 ± 1.7 | 15.5 ± 2.3 | 15.8 ± 2.1 | 13.6 ± 1.7 | 15.9 ± 2.4 |
| 40-49 | 30.5 ± 2.0 | 28.6 ± 2.2 | 27.8 ± 2.2 | 19.8 ± 2.8 | 23.5 ± 2.1 | 25.2 ± 1.8 | 22.4 ± 2.0 | 31.2 ± 2.6 | 26.9 ± 2.5 | 28.5 ± 2.2 | 26.9 ± 2.6 | 28.4 ± 2.7 |
| 50-59 | 42.0 ± 2.4 | 40.0 ± 2.9 | 44.1 ± 2.7 | 36 ± 3.8 | 35.9 ± 2.3 | 40.2 ± 2.4 | 41.0 ± 3.0 | 38 ± 2.2 | 38.7 ± 2.8 | 41.3 ± 2.7 | 36.8 ± 2.5 | 38.4 ± 2.3 |
| 60-69 | 43.8 ± 2.5 | 56.8 ± 3.6 | 53.9 ± 2.9 | 43.4 ± 3.9 | 43 ± 2.8 | 52.1 ± 2.4 | 52.5 ± 2.8 | 53.5 ± 3 | 55.3 ± 3.0 | 48.5 ± 3.3 | 45.5 ± 3.1 | 52.1 ± 2.7 |
| 70+ | 48.8 ± 4.2 | 52.5 ± 4.5 | 43.9 ± 5.1 | 51.9 ± 3.7 | 48.4 ± 3.3 | 58.5 ± 2.9 | 50.1 ± 3.5 | 58.9 ± 2.9 | 58.1 ± 2.9 | 59.0 ± 3.8 | 55.8 ± 3.1 | 61.7 ± 3.4 |
| Area of residence* |  |  |  |  |  |  |  |  |  |  |  |  |
| Urban area | 32.7 ± 1.4 | 33.5 ± 1.4 | 32.6 ± 1.4 | 27.3 ± 1.8 | 28.1 ± 1.1 | 31.8 ± 1.2 | 30.4 ± 1.2 | 32.6 ± 1.1 | 33.6 ± 1.4 | 33.0 ± 1.2 | 30.6 ± 1.4 | 32.6 ± 1.6 |
| Rural area | 31.3 ± 1.8 | 32.9 ± 2.9 | 29.9 ± 2.2 | 26.7 ± 3.6 | 28.0 ± 2.3 | 25.0 ± 1.6 | 26.2 ± 2.2 | 35.0 ± 2.2 | 27.6 ± 3.7 | 30.8 ± 2.5 | 26.5 ± 2.7 | 34.0 ± 3.2 |
| Income quartiles* |  |  |  |  |  |  |  |  |  |  |  |  |
| Highest | 33.2 ± 1.9 | 30.2 ± 2.2 | 31.8 ± 2.2 | 26.8 ± 2.6 | 25.8 ± 1.9 | 29.6 ± 1.8 | 27.2 ± 2.0 | 32.0 ± 2.3 | 30.4 ± 2.5 | 32.7 ± 2.7 | 27.7 ± 2.7 | 30.2 ± 2.7 |
| Upper middle | 29.9 ± 1.9 | 33.5 ± 2.3 | 28.7 ± 2.5 | 22.9 ± 2.5 | 29.2 ± 1.8 | 31.2 ± 2.0 | 30.1 ± 2.3 | 33.0 ± 2.3 | 34.5 ± 2.4 | 32.1 ± 2.4 | 34.7 ± 2.5 | 31.7 ± 2.3 |
| Lower middle | 34.2 ± 2.1 | 30.8 ± 2.1 | 30.6 ± 1.9 | 27.5 ± 2.7 | 28.4 ± 2.0 | 31.9 ± 2.0 | 29.6 ± 2.1 | 33.9 ± 2.2 | 32.9 ± 2.7 | 33.3 ± 2.4 | 28.2 ± 2.3 | 30.1 ± 2.4 |
| Lowest | 33.0 ± 2.1 | 37.5 ± 2.5 | 34.7 ± 2.3 | 32.3 ± 3.8 | 29.3 ± 1.9 | 28.8 ± 1.8 | 30.5 ± 2.2 | 31.9 ± 2.4 | 31.9 ± 2.4 | 31.7 ± 2.1 | 28.6 ± 2.4 | 39.3 ± 2.8 |
| Education levels* |  |  |  |  |  |  |  |  |  |  |  |  |
| Primary | 34.5±3.0 | 34.7±4.9 | 28.3±2.9 | 23.5 ± 3.0 | 36.8 ± 1.1 | 29.8 ± 2.6 | 43.9 ± 8.6 | 32.9 ± 5.1 | 40.7 ± 5.4 | 44.9 ± 4.7 | 29.9 ± 8.4 | 57.9 ± 8.4 |
| Middle | 31.5±2.5 | 33.7±2.5 | 33.1±1.8 | 23.1 ± 3.3 | 31.1 ± 4.4 | 26.7 ± 2.3 | 30.4 ± 5.4 | 30.6 ± 2.9 | 37.5 ± 8.7 | 39.8 ± 6.7 | 24.9 ± 4.2 | 40.7± 10.9 |
| High | 34.1±1.9 | 38.1±2.0 | 30.7±2.0 | 27.6 ± 3.2 | 29.5 ± 1.6 | 30.6 ± 1.6 | 30.5 ± 1.7 | 35.5 ± 1.8 | 35.6 ± 2.1 | 32.5 ±1.9 | 31.4 ± 2.1 | 35.0 ± 2.3 |
| University/college | 36.4±2.7 | 31.6±2.3 | 32.8±2.9 | 25.6 ± 2.5 | 28.5 ± 2.0 | 32.5 ± 1.7 | 28.9 ± 1.9 | 29.9 ± 1.7 | 28.7 ± 2.0 | 31.0 ± 2.0 | 29.4 ± 2.0 | 28.6 ± 2.0 |

Data are presented as mean ± SE. *age-standardized rate (standardized to the population in 2005).

**Supplementary Table 3.** Prevalence of hypertension in women, 1998‒2015

|  | 1998 | 2001 | 2005 | 2007 | 2008 | 2009 | 2010 | 2011 | 2012 | 2013 | 2014 | 2015 |
| --- | --- | --- | --- | --- | --- | --- | --- | --- | --- | --- | --- | --- |
| Prevalence | 27.0 ± 1.0 | 26.5 ± 1.2 | 25.4 ± 1.1 | 23.8 ± 1.5 | 26.4 ± 0.9 | 25.4 ± 1.0 | 27.7 ± 1.1 | 27.8 ± 1.1 | 29.8 ± 1.5 | 26.9 ± 1.1 | 26.2 ± 1.1 | 29.1 ± 1.1 |
| Prevalence (standardized) | 26.9 ± 0.8 | 25.4 ± 1.0 | 23.9 ± 0.9 | 21.8 ± 1.2 | 23.9 ± 0.7 | 22.2 ± 0.8 | 23.9 ± 0.7 | 23.7 ± 0.8 | 25.4 ± 1.1 | 22.2 ± 0.8 | 21 ± 0.7 | 23.1 ± 0.8 |
| Age (years) |  |  |  |  |  |  |  |  |  |  |  |  |
| 30-39 | 6.2 ± 0.8 | 5.4 ± 1.0 | 3.1 ± 0.7 | 1.5 ± 0.7 | 4.3 ± 0.9 | 2.9 ± 0.6 | 1.6 ± 0.5 | 3.4 ± 0.8 | 3.2 ± 0.9 | 3.7 ± 0.9 | 1.1 ± 0.4 | 1.6 ± 0.6 |
| 40-49 | 19.6 ± 1.5 | 15.2 ± 1.6 | 11.2 ± 1.5 | 11.4 ± 2.6 | 15.0 ± 1.4 | 11.0 ± 1.4 | 11.6 ± 1.4 | 10.8 ± 1.5 | 18.1 ± 2.2 | 10.7 ± 1.3 | 8.8 ± 1.5 | 13.0 ± 1.7 |
| 50-59 | 37.2 ± 1.9 | 33.2 ± 2.6 | 38.4 ± 2.9 | 31.1 ± 3.5 | 32.6 ± 2.1 | 30.5 ± 2.0 | 33.7 ± 2.2 | 29.7 ± 2.1 | 30.4 ± 2.3 | 30.6 ± 2.0 | 27.4 ± 2.2 | 29.5 ± 2.1 |
| 60-69 | 50.5 ± 2.5 | 57.5 ± 3.0 | 53.6 ± 3.0 | 48.2 ± 4.7 | 50.2 ± 2.1 | 49.1 ± 2.4 | 58.3 ± 3.1 | 57.1 ± 2.4 | 52.8 ± 2.8 | 48.8 ± 2.7 | 51.1 ± 2.4 | 51.5 ± 2.7 |
| 70+ | 63.4 ± 2.8 | 61.5 ± 2.9 | 61.3 ± 3.2 | 65.4 ± 3.8 | 61.5 ± 2.6 | 65.9 ± 2.3 | 68.2 ± 2.7 | 71.5 ± 2.7 | 71.6 ± 2.1 | 64.3 ± 3.0 | 68.7 ± 2.4 | 71.3 ± 2.4 |
| Area of residence* |  |  |  |  |  |  |  |  |  |  |  |  |
| Urban area | 27.3 ± 1.1 | 25.4 ± 1.1 | 23.8 ± 1.1 | 22.2 ± 1.4 | 23.7 ± 0.9 | 22.0 ± 0.9 | 24.2 ± 0.8 | 24.2 ± 0.8 | 24.5 ± 1.1 | 22.1 ± 0.9 | 20.9 ± 0.8 | 22.5 ± 0.8 |
| Rural area | 26.6 ± 1.3 | 27.2 ± 2.1 | 25.1 ± 1.7 | 21.3 ± 2.5 | 24.9 ± 1.3 | 23.1 ± 1.3 | 23.5 ± 1.6 | 23.4 ± 2.3 | 29.8 ± 3.2 | 23.1 ± 1.6 | 21.7 ± 2.0 | 26.7 ± 2.4 |
| Income quartiles* |  |  |  |  |  |  |  |  |  |  |  |  |
| Highest | 25.3 ± 1.5 | 22.7 ± 1.6 | 21.3 ± 1.6 | 22.6 ± 2.0 | 22.8 ± 1.4 | 21.3 ± 1.1 | 24.3 ± 1.8 | 21.8 ± 1.3 | 21.8 ± 1.4 | 19.6 ± 1.3 | 17.8 ± 1.2 | 20.5 ± 1.4 |
| Upper middle | 28.6 ± 1.5 | 27.4 ± 1.9 | 24.5 ± 1.8 | 19.5 ± 1.7 | 23.1 ± 1.4 | 20.2 ± 1.6 | 23.5 ± 1.6 | 22.7 ± 1.3 | 24.7 ± 1.7 | 21.6 ± 1.3 | 20.1 ± 1.3 | 20.2 ± 1.4 |
| Lower middle | 27.2 ± 1.5 | 25.4 ± 1.8 | 24.4 ± 1.6 | 20.9 ± 2.0 | 23.8 ± 1.5 | 24.9 ± 1.5 | 22.9 ± 1.3 | 25.7 ± 1.6 | 27.0 ± 1.8 | 26.3 ± 1.6 | 23.0 ± 1.5 | 25.1 ± 1.6 |
| Lowest | 27.2 ± 1.5 | 25.4 ± 1.9 | 25.7 ± 1.8 | 25.5 ± 2.7 | 25.8 ± 1.6 | 21.9 ± 1.3 | 25.7 ± 1.4 | 24.3 ± 1.6 | 26.8 ± 2.1 | 22.0 ± 1.7 | 23.5 ± 1.4 | 26.7 ± 1.4 |
| Education levels* |  |  |  |  |  |  |  |  |  |  |  |  |
| Primary | 29.3 ± 1.5 | 27.7 ± 2.8 | 30.8 ± 3.8 | 25.5 ± 2.2 | 29.1 ± 3.0 | 26.7 ±1.9 | 33.6 ± 4.4 | 26.4 ± 3.4 | 49.9 ± 4.6 | 40.8 ± 9.3 | 28.9 ± 5.0 | 36.1 ± 3.2 |
| Middle | 33.2 ± 1.8 | 33.6 ± 2.2 | 27.8 ± 2.9 | 17.2 ± 2.9 | 31.3 ± 3.1 | 18.1 ± 2.0 | 22.4 ± 2.3 | 30.5 ± 5.0 | 38.7 ± 6.4 | 32.9 ± 5.9 | 17.9 ± 1.9 | 24.7 ± 2.8 |
| High | 30.6 ± 1.9 | 25.3 ± 2.0 | 24.4 ± 2.1 | 19.4 ± 2.9 | 22.6 ± 2.0 | 21.6 ± 1.8 | 22.0 ± 1.7 | 22.7 ± 1.6 | 22.0 ± 1.6 | 19.9 ± 1.6 | 19.7 ± 1.4 | 20.1 ± 1.6 |
| University/college | 20.1 ± 2.7 | 24.8 ± 3.0 | 19.2 ± 3.3 | 4.5 ± 1.7 | 14.3 ± 3.0 | 14.7 ± 1.8 | 16.3 ± 3.0 | 14.8 ± 3.1 | 19.9 ± 2.0 | 19.5 ± 1.7 | 17.8 ± 1.7 | 15.6 ± 2.0 |

Data are presented as mean ± SE. *age-standardized rate (standardized to the population in 2005).

**Supplementary Table 4.** Awareness of hypertension, 1998‒2015

|  | 1998 | 2001 | 2005 | 2007-2009 | 2010-2012 | 2013-2015 |
| --- | --- | --- | --- | --- | --- | --- |
| Awareness | 23.5 ± 1.0 | 34.1 ± 1.5 | 57.1 ± 1.8 | 66.3 ± 1 | 65.9 ± 1 | 67.3 ± 0.9 |
| Awareness (standardized) | 24.7 ± 1.0 | 33.5 ± 1.4 | 57.1 ± 1.5 | 65.4 ± 0.9 | 63.2 ± 0.9 | 63.6 ± 0.9 |
| Age (years) |  |  |  |  |  |  |
| 30-39 | 10.4 ± 2.0 | 8.6 ± 2.5 | 16.0 ± 4.4 | 25.4 ± 2.9 | 21.1 ± 2.9 | 20.2 ± 3.2 |
| 40-49 | 18.1 ± 1.9 | 19.3 ± 2.8 | 37.0 ± 3.7 | 47.7 ± 2.2 | 41.4 ± 2.4 | 43.9 ± 2.5 |
| 50-59 | 26.3 ± 2.1 | 35.1 ± 2.8 | 63.9 ± 3.1 | 69.3 ± 1.8 | 65.7 ± 1.7 | 61.4 ± 1.8 |
| 60-69 | 30.8 ± 2.2 | 44.3 ± 3.2 | 72.6 ± 2.6 | 82.1 ± 1.4 | 78.0 ± 1.4 | 82.0 ± 1.2 |
| 70+ | 28.8 ± 2.7 | 45.5 ± 3.4 | 69.6 ± 3.0 | 77.6 ± 1.3 | 85.3 ± 1.1 | 86.0 ± 0.9 |
| Area of residence^*^ |  |  |  |  |  |  |
| Urban area | 24.2 ± 1.3 | 33.1 ± 1.7 | 57.6 ± 1.7 | 65.4 ± 1.0 | 63.2 ± 1.1 | 63.9 ± 1.0 |
| Rural area | 25.0 ± 1.6 | 35.8 ± 2.8 | 56.1 ± 3.5 | 65.3 ± 1.7 | 63.7 ± 1.8 | 62.2 ± 1.8 |
| Income quartiles^*^ |  |  |  |  |  |  |
| Highest | 26.0 ± 2.1 | 35.2 ± 2.4 | 56.3 ± 3.0 | 63.1 ± 1.6 | 63.1 ± 1.7 | 65.1 ± 1.6 |
| Upper middle | 21.1 ± 1.9 | 34.1 ± 2.8 | 57.4 ± 2.6 | 64.2 ± 1.6 | 61.3 ± 1.6 | 62.4 ± 1.7 |
| Lower middle | 26.5 ± 2.2 | 33.5 ± 2.8 | 57.9 ± 3.2 | 64.2 ± 1.7 | 64.0 ± 1.5 | 60.4 ± 1.5 |
| Lowest | 25.4 ± 2.0 | 32.3 ± 2.6 | 56.6 ± 3.0 | 70.6 ± 1.7 | 64.4 ± 1.7 | 66.4 ± 1.8 |
| Education levels^*^ |  |  |  |  |  |  |
| Primary | 27.2 ± 4.0 | 23.6 ± 2.5 | 66.8 ± 2.9 | 65.6 ± 3.1 | 71.1 ± 3.2 | 65.3 ± 3.4 |
| Middle | 22.5 ± 2.6 | 29.1 ± 4.4 | 60.8 ± 3.8 | 62.9 ± 2.0 | 64.0 ± 2.6 | 60.0 ± 2.5 |
| High | 17.8 ± 2.0 | 22.5 ± 2.6 | 61.0 ± 2.3 | 67.1 ± 1.5 | 63.5 ± 1.4 | 65.2 ± 1.5 |
| University/college | 16.6 ± 2.8 | 21.3 ± 3.2 | 57.0 ± 6.3 | 67.6 ± 2.2 | 64.0 ± 1.8 | 60.8 ± 1.9 |

Data are presented as mean ± SE. *age-standardized rate (standardized to the population in 2005).

**Supplementary Table 5.** Awareness of hypertension in men, 1998‒2015

|  | 1998 | 2001 | 2005 | 2007-2009 | 2010-2012 | 2013-2015 |
| --- | --- | --- | --- | --- | --- | --- |
| Awareness | 17.3 ± 1.3 | 26.8 ± 1.8 | 48.4 ± 2.5 | 58.4 ± 1.3 | 56.9 ± 1.4 | 60.1 ± 1.4 |
| Awareness (standardized) | 19.7 ± 1.4 | 28.9 ± 1.9 | 53.0 ± 2.2 | 61.7 ± 1.2 | 59.6 ± 1.2 | 61.9 ± 1.2 |
| Age (years) |  |  |  |  |  |  |
| 30-39 | 9.1 ± 2.4 | 7.0 ± 2.8 | 15.4 ± 4.9 | 24.1 ± 3.4 | 17.6 ± 3.1 | 20.7 ± 3.6 |
| 40-49 | 15.0 ± 2.2 | 18.8 ± 3.4 | 34.9 ± 4.5 | 42.5 ± 3.0 | 36.5 ± 3.0 | 40.8 ± 3.1 |
| 50-59 | 18.5 ± 2.5 | 32.1 ± 3.8 | 60.4 ± 4.0 | 64.6 ± 2.5 | 61.9 ± 2.5 | 58.5 ± 2.5 |
| 60-69 | 25.8 ± 3.3 | 31.8 ± 4.1 | 65.0 ± 4.2 | 79.5 ± 2.2 | 74.4 ± 2.1 | 82.3 ± 1.7 |
| 70+ | 24.0 ± 3.9 | 42.5 ± 5.8 | 65.7 ± 6.3 | 74.3 ± 2.2 | 83.4 ± 1.7 | 84.5 ± 1.5 |
| Area of residence* |  |  |  |  |  |  |
| Urban area | 19.4 ± 2.0 | 27.0 ± 2.1 | 54.4 ± 2.6 | 61.9 ± 1.4 | 60.2 ± 1.3 | 62.4 ±1.3 |
| Rural area | 18.8 ± 1.7 | 35.6 ± 4.3 | 47.5 ± 4.3 | 61.1 ± 2.5 | 58.7 ± 2.8 | 60.2 ±2.4 |
| Income quartiles* |  |  |  |  |  |  |
| Highest | 22.3 ± 3.2 | 28.7 ± 3.4 | 51.4 ± 4.2 | 57.9 ± 2.3 | 58.0 ± 2.4 | 61.0 ± 2.3 |
| Upper middle | 18.2 ± 2.4 | 35.7 ± 3.2 | 55.6 ± 4.0 | 61.1 ± 2.3 | 56.4 ± 2.2 | 61.0 ± 2.3 |
| Lower middle | 20.2 ± 3.4 | 28.6 ± 3.7 | 53.2 ± 4.9 | 59.5 ± 2.2 | 60.0 ± 2.0 | 58.1 ± 2.0 |
| Lowest | 18.3 ± 2.6 | 28.2 ± 3.5 | 53.0 ± 4.3 | 69.3 ± 2.0 | 63.9 ± 2.3 | 67.1 ± 2.2 |
| Education levels* |  |  |  |  |  |  |
| Primary | 19.0 ± 5.2 | 22.6 ± 4.8 | 43.7 ± 4.2 | 56.0 ± 2.7 | 63.6 ± 4.8 | 61.3 ± 4.2 |
| Middle | 21.0 ± 3.9 | 18.2 ± 2.9 | 48.3 ± 5.1 | 58.1 ± 2.6 | 57.5 ± 2.9 | 54.2 ± 3.4 |
| High | 16.6 ± 2.1 | 22.6 ± 3.2 | 60.0 ± 2.8 | 65.3 ± 1.8 | 60.0 ± 1.9 | 64.4 ± 1.9 |
| University/college | 15.9 ± 3.0 | 21.8 ± 3.0 | 54.7 ± 6.5 | 66.5 ± 2.4 | 64.7 ± 2.1 | 60.5 ± 2.4 |

Data are presented as mean ± SE. *age-standardized rate (standardized to the population in 2005).

**Supplementary Table 6.** Awareness of hypertension in women, 1998‒2015

|  | 1998 | 2001 | 2005 | 2007-2009 | 2010-2012 | 2013-2015 |
| --- | --- | --- | --- | --- | --- | --- |
| Awareness | 30.0 ± 1.6 | 40.8 ± 2.1 | 67.2 ± 2.1 | 75.0 ± 1.1 | 75.7 ± 1.1 | 75.4 ± 1.0 |
| Awareness (standardized) | 29.6 ± 1.5 | 37.5 ± 1.9 | 61.5 ± 2.0 | 70.3 ± 1.3 | 69.2 ± 1.4 | 66.0 ± 1.3 |
| Age (years) |  |  |  |  |  |  |
| 30-39 | 14.3 ± 4.3 | 12.2 ± 5.7 | 18.5 ± 9.9 | 31.1 ± 6.9 | 40.2 ± 8.3 | 17.0 ± 6.7 |
| 40-49 | 23.1 ± 3.5 | 19.9 ± 4.4 | 42.7 ± 6.4 | 57.7 ± 3.3 | 51.5 ± 3.8 | 52.0 ± 4.1 |
| 50-59 | 34.8 ± 3.4 | 37.8 ± 4.1 | 67.9 ± 4.6 | 74.8 ± 2.2 | 70.3 ± 2.1 | 65.1 ± 2.4 |
| 60-69 | 34.4 ± 3.3 | 54.3 ± 3.9 | 79.2 ± 2.9 | 84.4 ± 1.6 | 81.1 ± 1.7 | 81.8 ± 1.7 |
| 70+ | 30.7 ± 3.6 | 47.1 ± 4.0 | 71.2 ± 3.4 | 79.3 ± 1.5 | 86.2 ± 1.5 | 86.9 ± 1.2 |
| Area of residence* |  |  |  |  |  |  |
| Urban area | 29.0 ± 2.0 | 38.4 ± 2.4 | 60.7 ± 2.1 | 70.2 ± 1.5 | 69.1 ± 1.7 | 66.0 ± 1.5 |
| Rural area | 30.6 ± 2.6 | 35.8 ± 3.0 | 57.2 ± 3.0 | 71.4 ± 2.5 | 69.2 ± 2.4 | 65.3 ± 2.8 |
| Income quartiles* |  |  |  |  |  |  |
| Highest | 30.3 ± 3.1 | 41.4 ± 4.3 | 64.4 ± 4.0 | 68.3 ± 2.5 | 70.0 ± 2.3 | 68.7 ± 2.2 |
| Upper middle | 24.3 ± 2.8 | 35.0 ± 4.4 | 58.8 ± 4.1 | 68.3 ± 2.5 | 69.6 ± 2.4 | 65.4 ± 2.5 |
| Lower middle | 31.0 ± 3.1 | 38.1 ± 3.8 | 60.9 ± 4.1 | 71.7 ± 2.8 | 70.5 ± 2.6 | 65.7 ± 2.8 |
| Lowest | 31.6 ± 3.2 | 34.3 ± 3.8 | 59.7 ± 4.4 | 72.7 ± 2.7 | 62.5 ± 2.7 | 62.0 ± 2.8 |
| Education levels* |  |  |  |  |  |  |
| Primary | 32.4 ± 5.6 | 24.0 ± 2.6 | 72.5 ± 3.4 | 75.1 ± 2.0 | 76.4 ± 3.7 | 73.7 ± 3.6 |
| Middle | 25.3 ± 3.8 | 38.9 ± 6.4 | 69.5 ± 5.2 | 69.4 ± 3.2 | 70.0 ± 3.5 | 67.0 ± 4.1 |
| High | 20.7 ± 3.9 | 24.3 ± 4.0 | 65.0 ± 4.1 | 69.9 ± 2.5 | 71.4 ± 2.2 | 66.9 ± 2.2 |
| University/college | 33.0 ± 8.0 | 20.8 ± 6.4 | 69.2 ± 7.5 | 75.0 ± 4.8 | 60.3 ± 5.0 | 61.8 ± 3.5 |

Data are presented as mean ± SE. *age-standardized rate

**Supplementary Figure 3.** Age-standardized awareness of hypertension (A) in total study subjects, and according to (B) the area of residence, (C) income quartiles, and (D) education levels

| 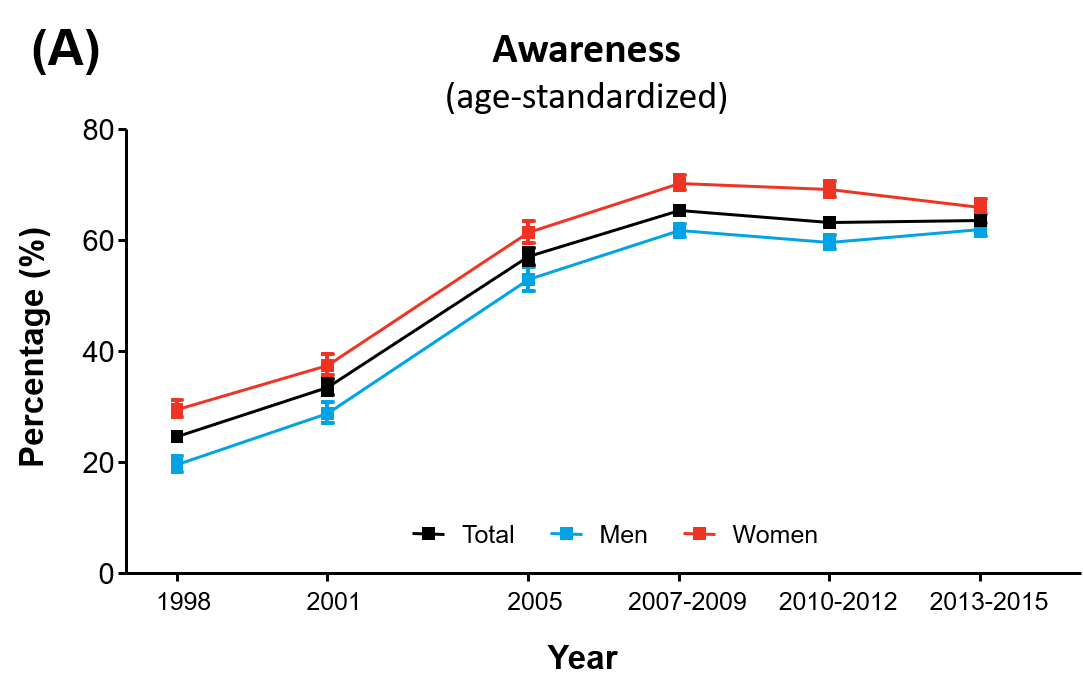 | 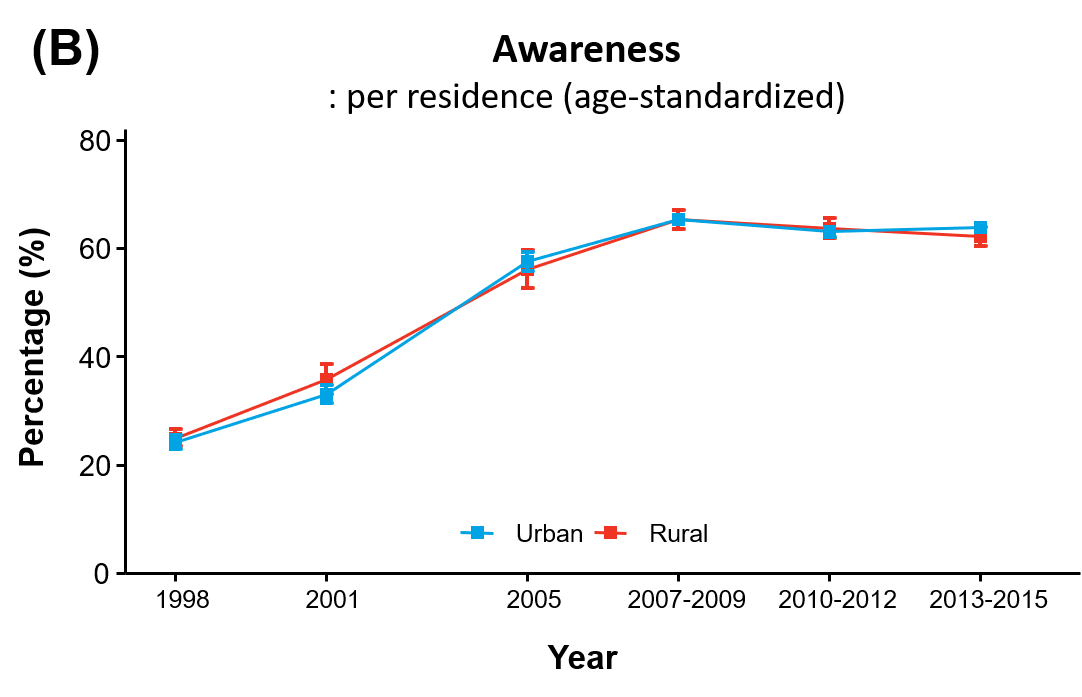 |
| --- | --- |
| 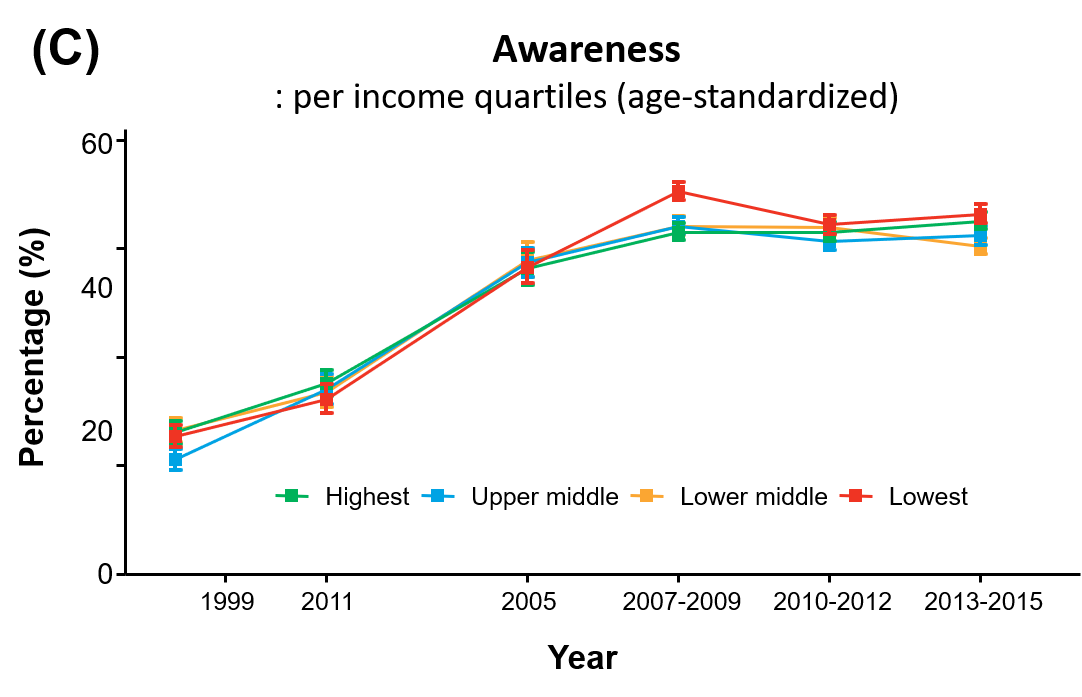 | 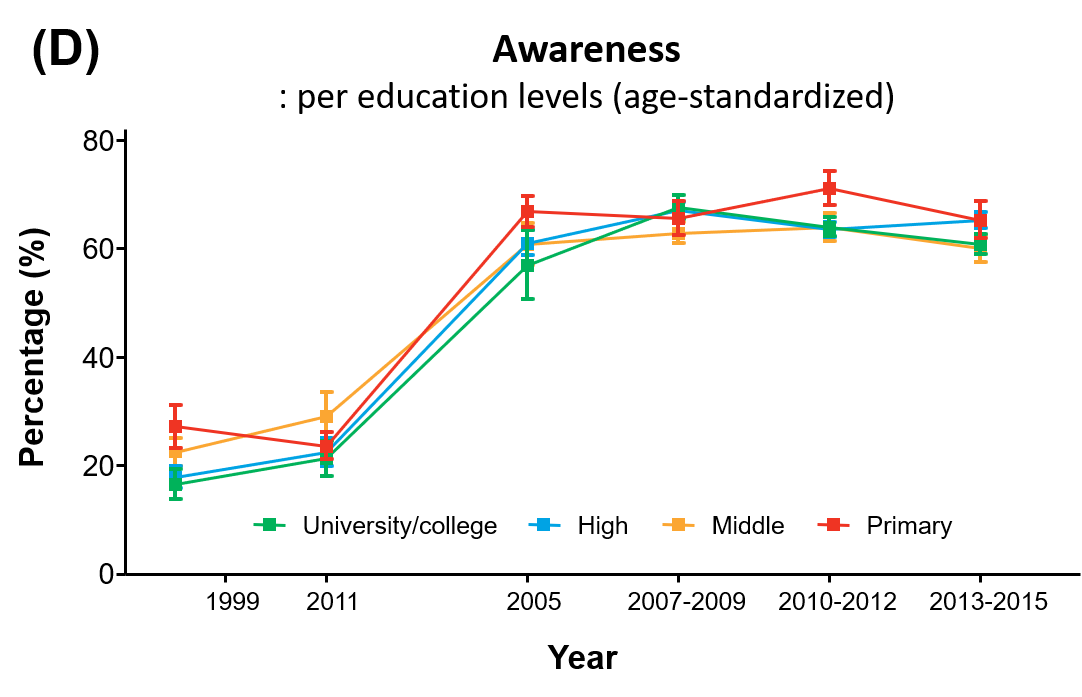 |

**Supplementary Table 7.** Treatment of hypertension, 1998‒2015

|  | 1998 | 2001 | 2005 | 2007-2009 | 2010-2012 | 2013-2015 |
| --- | --- | --- | --- | --- | --- | --- |
| Treatment | 20.4 ± 1.0 | 32.7 ± 1.5 | 49.6 ± 1.8 | 60.3 ± 1.0 | 60.7 ± 1.0 | 63.6 ± 0.9 |
| Treatment (standardized) | 22.0 ± 1.0 | 31.9 ± 1.4 | 49.5 ± 1.5 | 59.2 ± 0.9 | 57.6 ± 0.9 | 59.7 ± 0.8 |
| Age (years) |  |  |  |  |  |  |
| 30-39 | 5.0 ± 1.5 | 8.3 ± 2.7 | 8.9 ± 3.5 | 15.4 ± 2.4 | 13.4 ± 2.3 | 15.2 ± 2.8 |
| 40-49 | 14.0 ± 1.9 | 18.2 ± 2.4 | 27.6 ± 3.5 | 38.8 ± 2.3 | 34.2 ± 2.2 | 39.5 ± 2.6 |
| 50-59 | 22.0 ± 2.0 | 30.7 ± 3.0 | 54.6 ± 3.1 | 62.5 ± 1.8 | 58.4 ± 1.7 | 56.2 ± 1.8 |
| 60-69 | 29.3 ± 2.4 | 45.2 ± 3.1 | 65.8 ± 2.8 | 78.6 ± 1.5 | 74.4 ± 1.5 | 79.3 ± 1.3 |
| 70+ | 29.6 ± 2.7 | 43.0 ± 3.5 | 65.5 ± 3.2 | 73.5 ± 1.3 | 82.7 ± 1.2 | 83.4 ± 1.0 |
| Area of residence^*^ |  |  |  |  |  |  |
| Urban area | 23.1 ± 1.2 | 31.7 ± 1.7 | 49.6 ± 1.7 | 59.4 ± 1.1 | 57.2 ± 1.0 | 59.8 ± 0.9 |
| Rural area | 19.5 ± 1.5 | 33.3 ± 2.5 | 48.9 ± 3.4 | 58.6 ± 1.5 | 60.0 ± 1.9 | 59.4 ± 1.8 |
| Income quartiles^*^ |  |  |  |  |  |  |
| Highest | 22.1 ± 2.1 | 33.2 ± 3.3 | 46.0 ± 2.9 | 57.8 ± 1.7 | 56.6 ± 1.6 | 60.5 ± 1.6 |
| Upper middle | 19.2 ± 1.8 | 30.7 ± 2.8 | 53.6 ± 2.5 | 57.0 ± 1.6 | 56.6 ± 1.6 | 59.1 ± 1.6 |
| Lower middle | 21.9 ± 2.5 | 31.6 ± 2.5 | 50.9 ± 3.2 | 58.8 ± 1.8 | 58.9 ± 1.5 | 57.0 ± 1.6 |
| Lowest | 23.8 ± 1.9 | 31.8 ± 2.5 | 48.9 ± 3.1 | 63.7 ± 1.8 | 58.4 ± 1.7 | 62.5 ± 1.8 |
| Education levels^*^ |  |  |  |  |  |  |
| Primary | 25.0 ± 1.8 | 33.5 ± 2.8 | 50.2 ± 2.7 | 60.2 ± 3.1 | 65.0 ± 2.9 | 61.7 ± 3.3 |
| Middle | 19.8 ± 2.9 | 37.0 ± 3.2 | 44.3 ± 3.4 | 56.9 ± 2.1 | 58.3 ± 2.6 | 56.1 ± 2.6 |
| High | 21.2 ± 3.7 | 31.4 ± 3.4 | 52.7 ± 2.4 | 61.0 ± 1.6 | 58.4 ± 1.5 | 61.5 ± 1.5 |
| University/college | 23.3 ± 3.7 | 30.4 ± 4.2 | 51.8 ± 5.8 | 63.2 ± 2.4 | 56.7 ± 1.9 | 57.6 ± 1.9 |

Data are presented as mean ± SE. *age-standardized rate (standardized to the population in 2005).

**Supplementary Table 8.** Treatment of hypertension in men, 1998‒2015

|  | 1998 | 2001 | 2005 | 2007-2009 | 2010-2012 | 2013-2015 |
| --- | --- | --- | --- | --- | --- | --- |
| Treatment | 13.8 ± 1.2 | 25.2 ± 1.7 | 39.4 ± 2.3 | 51.1 ± 1.4 | 51.4 ± 1.4 | 55.4 ± 1.4 |
| Treatment (standardized) | 16.7 ± 1.3 | 26.8 ± 1.8 | 44.4 ± 2.1 | 54.9 ± 1.2 | 54.3 ± 1.2 | 57.4 ± 1.1 |
| Age (years) |  |  |  |  |  |  |
| 30-39 | 3.7 ± 1.5 | 6.9 ± 3.1 | 9.4 ± 4.0 | 13.7 ± 2.7 | 10.6 ± 2.4 | 15.0 ± 3.0 |
| 40-49 | 11.0 ± 2.1 | 14.8 ± 2.9 | 24.2 ± 3.8 | 31.2 ± 2.9 | 29.5 ± 2.8 | 36.0 ± 3.1 |
| 50-59 | 17.5 ± 2.6 | 25.7 ± 3.2 | 49.1 ± 4.0 | 58.2 ± 2.5 | 55.1 ± 2.6 | 52.6 ± 2.5 |
| 60-69 | 20.2 ± 3.2 | 35.3 ± 4.5 | 57.4 ± 4.3 | 75.5 ± 2.4 | 71.8 ± 2.1 | 79.4 ± 1.8 |
| 70+ | 23.9 ± 4.4 | 40.6 ± 5.5 | 60.5 ± 6.2 | 70.1 ± 2.3 | 79.8 ± 1.9 | 80.3 ± 1.7 |
| Area of residence* |  |  |  |  |  |  |
| Urban area | 18.9 ±1.9 | 26.3 ± 2.2 | 45.1 ± 2.6 | 55.3 ± 1.5 | 54.5 ± 1.3 | 57.5 ± 1.3 |
| Rural area | 12.4 ±1.5 | 29.7 ± 3.3 | 40.0 ± 3.0 | 54.1 ± 2.1 | 54.7 ± 2.9 | 56.9 ± 2.4 |
| Income quartiles* |  |  |  |  |  |  |
| Highest | 15.7 ± 2.8 | 21.6 ± 3.2 | 39.6 ± 3.8 | 52.6 ± 2.4 | 51.9 ± 2.4 | 57.8 ± 2.3 |
| Upper middle | 17.0 ± 2.6 | 27.9 ± 3.9 | 52.6 ± 3.9 | 52.4 ± 2.3 | 51.7 ± 2.1 | 56.5 ± 2.3 |
| Lower middle | 15.6 ± 3.0 | 27.3 ± 3.8 | 45.5 ± 4.5 | 54.0 ± 2.3 | 55.6 ± 2.1 | 54.1 ± 2.1 |
| Lowest | 17.5 ± 2.7 | 31.5 ± 4.0 | 41.9 ± 4.2 | 61.7 ± 2.4 | 57.9 ± 2.3 | 62.3 ± 2.3 |
| Education levels* |  |  |  |  |  |  |
| Primary | 15.5 ± 2.4 | 28.4 ± 4.3 | 36.0 ± 3.5 | 49.2 ± 2.7 | 55.6 ± 3.4 | 55.6 ± 4.0 |
| Middle | 14.2 ± 3.3 | 23.7 ± 4.3 | 38.2 ± 5.2 | 51.9 ± 2.6 | 52.7 ± 2.8 | 49.3 ± 3.4 |
| High | 18.2 ± 4.1 | 29.4 ± 4.1 | 51.4 ± 2.8 | 57.5 ± 1.9 | 55.3 ± 1.9 | 60.4 ± 1.9 |
| University/college | 23.5 ± 4.1 | 29.6 ± 4.6 | 48.8 ± 5.6 | 62.3 ± 2.6 | 58.5 ± 2.2 | 56.8 ± 2.3 |

Data are presented as mean ± SE. *age-standardized rate (standardized to the population in 2005).

**Supplementary Table 9.** Treatment of hypertension in women, 1998‒2015

|  | 1998 | 2001 | 2005 | 2007-2009 | 2010-2012 | 2013-2015 |
| --- | --- | --- | --- | --- | --- | --- |
| Treatment | 27.5 ± 1.5 | 39.5 ± 2.2 | 61.4 ± 2.4 | 70.3 ± 1.2 | 70.7 ± 1.1 | 72.7 ± 1.0 |
| Treatment (standardized) | 26.6 ± 1.5 | 36.4 ± 2.1 | 55.0 ± 2.3 | 65.3 ± 1.3 | 63.0 ± 1.3 | 63.2 ± 1.3 |
| Age (years) |  |  |  |  |  |  |
| 30-39 | 8.8 ± 3.6 | 11.4 ± 5.6 | 6.7 ± 6.5 | 22.9 ± 6.1 | 28.9 ± 7.5 | 17.0 ± 6.7 |
| 40-49 | 18.7 ± 3.6 | 22.6 ± 4.3 | 36.5 ± 6.4 | 53.5 ± 3.4 | 43.9 ± 3.8 | 48.5 ± 4.2 |
| 50-59 | 26.9 ± 3.1 | 35.2 ± 4.8 | 61.1 ± 4.8 | 67.7 ± 2.4 | 62.4 ± 2.1 | 60.9 ± 2.5 |
| 60-69 | 35.8 ± 3.3 | 53.2 ± 3.8 | 73.0 ± 3.5 | 81.2 ± 1.7 | 76.7 ± 1.9 | 79.2 ± 1.7 |
| 70+ | 31.8 ± 3.4 | 44.2 ± 4.2 | 67.5 ± 3.5 | 75.2 ± 1.6 | 84.2 ± 1.5 | 85.2 ± 1.3 |
| Area of residence* |  |  |  |  |  |  |
| Urban area | 27.3 ± 1.9 | 36.9 ± 2.6 | 54.4 ± 2.4 | 65.4 ± 1.5 | 62.0 ± 1.6 | 63.0 ± 1.5 |
| Rural area | 25.1 ± 2.2 | 36.0 ± 2.8 | 52.8 ± 3.8 | 64.9 ± 2.4 | 65.7 ± 2.5 | 63.6 ± 2.9 |
| Income quartiles* |  |  |  |  |  |  |
| Highest | 26.8 ± 3.1 | 44.7 ± 5.5 | 55.5 ± 4.5 | 64.1 ± 2.4 | 63.5 ± 2.2 | 63.8 ± 2.3 |
| Upper middle | 21.4 ± 2.6 | 35.6 ± 4.3 | 55.3 ± 4.2 | 63.9 ± 2.5 | 64.4 ± 2.4 | 63.6 ± 2.4 |
| Lower middle | 26.8 ± 3.0 | 35.3 ± 3.6 | 55.4 ± 4.1 | 66.9 ± 2.9 | 63.3 ± 2.6 | 63.1 ± 2.8 |
| Lowest | 30.1 ± 2.9 | 30.1 ± 3.3 | 53.8 ± 4.4 | 66.6 ± 2.5 | 56.5 ± 2.2 | 60.4 ± 2.8 |
| Education levels* |  |  |  |  |  |  |
| Primary | 29.1 ± 2.3 | 35.4 ± 3.0 | 59.2 ± 3.6 | 70.7 ± 2.1 | 73.7 ± 3.7 | 71.9 ± 3.6 |
| Middle | 26.4 ± 4.9 | 48.5 ± 4.6 | 52.8 ± 5.3 | 63.7 ± 3.2 | 63.8 ± 3.5 | 64.4 ± 4.1 |
| High | 30.5 ± 7.3 | 37.6 ± 4.6 | 57.1 ± 4.8 | 67.2 ± 2.5 | 65.5 ± 2.3 | 64.0 ± 2.3 |
| University/college | 37.7 ± 6.5 | 37.4 ± 8.2 | 67.8 ± 7.4 | 69.2 ± 5.5 | 49.3 ± 4.6 | 60.4 ± 3.5 |

Data are presented as mean ± SE. *age-standardized rate

**Supplementary Figure 4.** Age-standardized rate of hypertension treatment (A) in total study subjects, and according to (B) the area of residence, (C) income quartiles, and (D) education levels

| 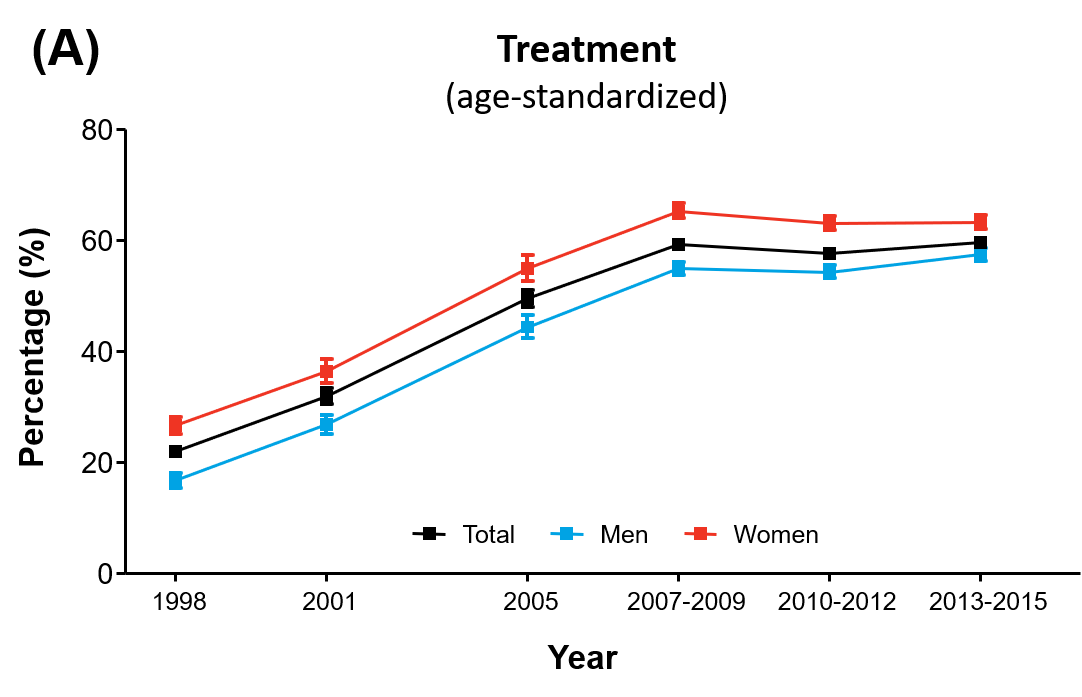 | 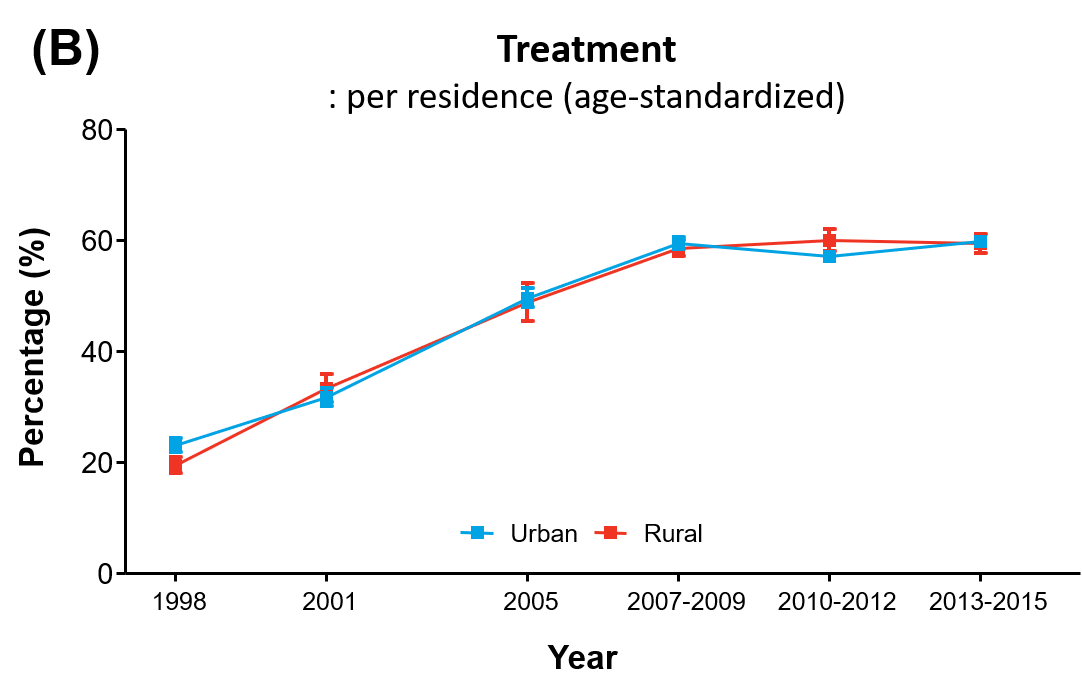 |
| --- | --- |
| 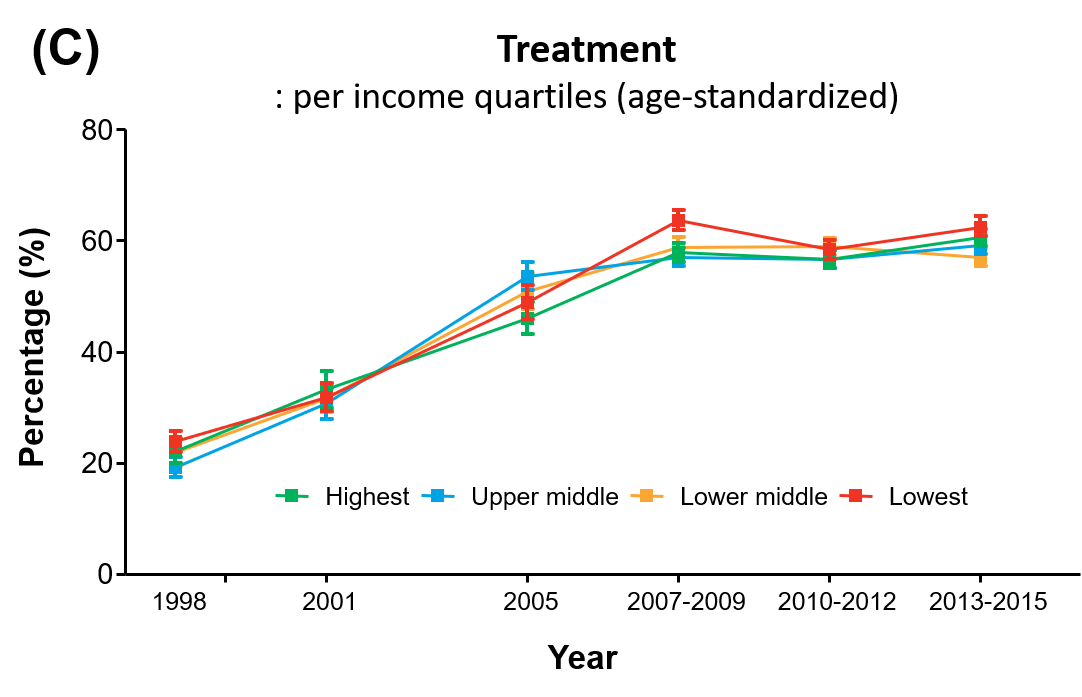 | 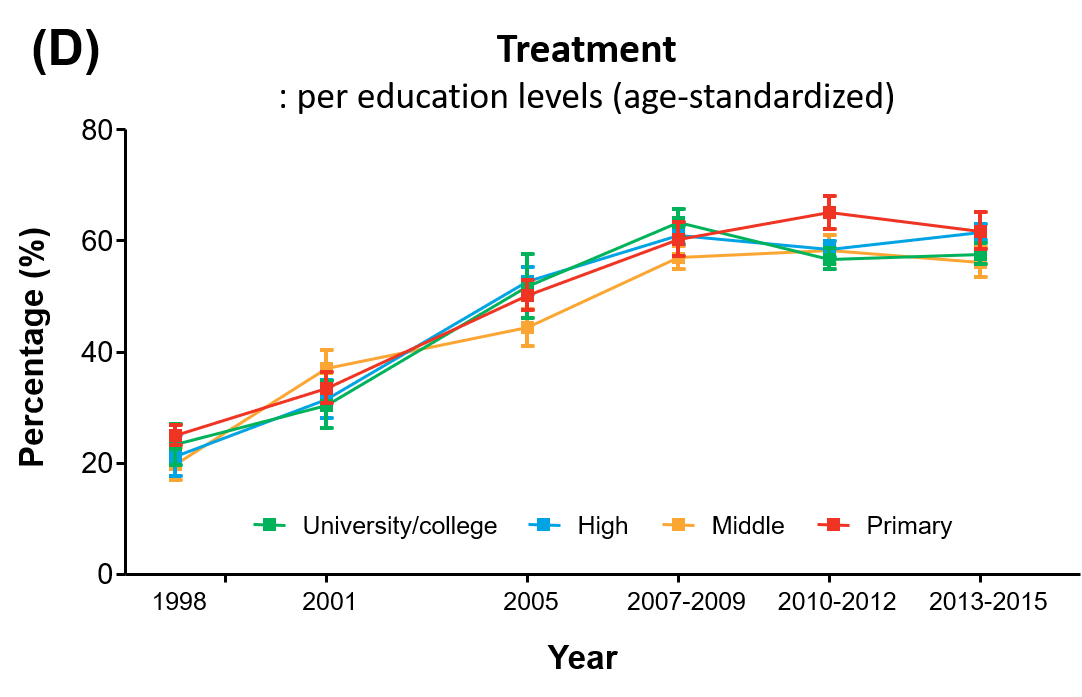 |

**Supplementary Table 10.** Adequate control of hypertension among hypertensive subjects, 1998‒2015

|  | 1998 | 2001 | 2005 | 2007-2009 | 2010-2012 | 2013-2015 |
| --- | --- | --- | --- | --- | --- | --- |
| Control | 4.9 ± 0.6 | 12.3 ± 1.0 | 27.2 ± 1.7 | 42.1 ± 1.1 | 42.5 ± 0.9 | 46.2 ± 1.0 |
| Control (standardized) | 5.1 ± 0.6 | 12.1 ± 1.0 | 27.2 ± 1.6 | 41.4 ± 1.1 | 40.5 ± 0.9 | 43.5 ± 0.9 |
| Age (years) |  |  |  |  |  |  |
| 30-39 | 1.3 ± 0.8 | 4.6 ± 2.0 | 6.4 ± 3.0 | 9.9 ± 2.1 | 9.2 ± 2.1 | 9.8 ± 2.2 |
| 40-49 | 4.8 ± 1.1 | 7.1 ± 1.5 | 14.9 ± 2.9 | 27.9 ± 2.2 | 24.3 ± 2.0 | 29.8 ± 2.4 |
| 50-59 | 5.9 ± 1.1 | 12.3 ± 2.1 | 32.7 ± 3.2 | 43.8 ± 2.0 | 42.0 ± 1.7 | 40.1 ± 1.8 |
| 60-69 | 6.2 ± 1.4 | 16.1 ± 2.2 | 35.2 ± 3.0 | 56.3 ± 1.9 | 51.8 ± 1.6 | 59.6 ± 1.6 |
| 70+ | 4.8 ± 1.2 | 15.7 ± 2.7 | 33.0 ± 3.4 | 49.4 ± 1.7 | 57.0 ± 1.5 | 59.3 ± 1.4 |
| Area of residence^*^ |  |  |  |  |  |  |
| Urban area | 5.5 ± 0.8 | 11.8 ± 1.2 | 27.1 ± 1.9 | 41.6 ± 1.3 | 40.0 ± 1.0 | 43.9 ± 1.0 |
| Rural area | 4.3 ± 0.9 | 13.7 ± 2.0 | 26.7 ± 3.1 | 40.9 ± 1.7 | 42.8 ± 1.9 | 41.8 ± 2.1 |
| Income quartiles^*^ |  |  |  |  |  |  |
| Highest | 4.0 ± 1.0 | 12.5 ± 1.9 | 26.2 ± 2.8 | 38.3 ± 1.9 | 39.2 ± 1.6 | 44.9 ± 1.6 |
| Upper middle | 4.6 ± 1.0 | 10.0 ± 1.9 | 26.9 ± 2.4 | 40.0 ± 1.7 | 40.7 ± 1.6 | 41.5 ± 1.7 |
| Lower middle | 5.2 ± 1.3 | 11.3 ± 2.0 | 30.0 ± 3.0 | 42.6 ± 1.8 | 41.7 ± 1.7 | 43.4 ± 1.6 |
| Lowest | 6.4 ± 1.2 | 13.2 ± 2.1 | 25.4 ± 3.1 | 45.3 ± 2.0 | 40.4 ± 1.7 | 44.9 ± 1.8 |
| Education levels^*^ |  |  |  |  |  |  |
| Primary | 4.5 ± 0.7 | 13.1 ± 1.9 | 29.4 ± 3.2 | 38.9 ± 1.7 | 47.8 ± 3.0 | 44.5 ± 2.7 |
| Middle | 5.2 ± 1.9 | 17.8 ± 3.2 | 26.3 ± 3.2 | 39.4 ± 2.0 | 40.4 ± 2.1 | 42.7 ± 2.6 |
| High | 3.6 ± 0.9 | 11.8 ± 2.2 | 26.2 ± 2.9 | 45.9 ± 1.9 | 40.4 ± 1.6 | 47.2 ± 1.6 |
| University/college | 6.3 ± 2.3 | 8.8 ± 3.1 | 28.9 ± 4.9 | 46.9 ± 3.0 | 39.9 ± 2.1 | 41.7 ± 2.1 |

Data are presented as mean ± SE. *age-standardized rate (standardized to the population in 2005).

**Supplementary Table 11.** Adequate control of hypertension among hypertensive subjects in men, 1998‒2015

|  | 1998 | 2001 | 2005 | 2007-2009 | 2010-2012 | 2013-2015 |
| --- | --- | --- | --- | --- | --- | --- |
| Control | 3.3 ± 0.6 | 7.6 ± 1.2 | 20.1 ± 2.0 | 35.4 ± 1.4 | 37.3 ± 1.3 | 40.4 ± 1.3 |
| Control (standardized) | 3.6 ± 0.7 | 8.1 ± 1.3 | 22.0 ± 2.0 | 38.0 ± 1.4 | 39.5 ± 1.2 | 42.1 ± 1.2 |
| Age (years) |  |  |  |  |  |  |
| 30-39 | 1.1 ± 0.8 | 2.9 ± 1.7 | 6.3 ± 3.4 | 8.7 ± 2.2 | 7.6 ± 2.2 | 9.7 ± 2.3 |
| 40-49 | 3.1 ± 1.1 | 5.4 ± 2.0 | 10.6 ± 2.7 | 21.6 ± 2.6 | 20.8 ± 2.4 | 24.2 ± 2.7 |
| 50-59 | 4.7 ± 1.5 | 9.1 ± 2.3 | 28.3 ± 4.5 | 40.8 ± 2.6 | 39.9 ± 2.6 | 35.9 ± 2.4 |
| 60-69 | 3.5 ± 1.4 | 8.7 ± 2.1 | 30.7 ± 4.0 | 53.2 ± 2.6 | 51.9 ± 2.3 | 61.7 ± 2.3 |
| 70+ | 4.1 ± 1.8 | 11.6 ± 3.6 | 22.1 ± 5.1 | 46.9 ± 2.6 | 59.5 ± 2.4 | 61.2 ± 2.3 |
| Area of residence* |  |  |  |  |  |  |
| Urban area | 3.7 ± 0.9 | 7.9 ± 1.4 | 22.3 ± 2.5 | 38.2 ± 1.7 | 39.4 ± 1.3 | 43.0 ± 1.3 |
| Rural area | 3.2 ± 1.0 | 9.2 ± 2.7 | 18.8 ± 2.8 | 37.4 ± 2.3 | 40.6 ± 2.7 | 38.4 ±2.6 |
| Income quartiles* |  |  |  |  |  |  |
| Highest | 3.3 ± 1.3 | 7.8 ± 2.1 | 20.2 ± 3.7 | 33.5 ± 2.5 | 37.8 ± 2.2 | 44.1 ± 2.2 |
| Upper middle | 3.4 ± 1.3 | 5.4 ± 2.1 | 25.9 ± 3.5 | 36.5 ± 2.4 | 40.8 ± 2.2 | 39.5 ± 2.3 |
| Lower middle | 3.3 ± 1.4 | 6.5 ± 1.9 | 22.6 ± 4.2 | 38.8 ± 2.4 | 39.5 ± 2.2 | 41.0 ± 2.0 |
| Lowest | 4.3 ± 1.3 | 11.3 ± 3.0 | 19.8 ± 3.7 | 44.8 ± 2.6 | 40.5 ± 2.4 | 45.0 ± 2.4 |
| Education levels* |  |  |  |  |  |  |
| Primary | 2.6 ± 0.9 | 9.7 ± 3.6 | 17.6 ± 2.9 | 33.4 ± 2.8 | 43.0 ± 3.5 | 38.5 ± 3.1 |
| Middle | 3.3 ± 1.7 | 6.8 ± 3.0 | 21.6 ± 5.0 | 33.1 ± 2.6 | 38.2 ± 2.8 | 37.6 ± 2.9 |
| High | 2.2 ± 0.7 | 10.6 ± 2.5 | 19.0 ± 2.8 | 41.3 ± 2.2 | 39.1 ± 2.0 | 46.9 ± 1.9 |
| University/college | 6.3 ± 2.4 | 7.5 ± 2.6 | 27.3 ± 5.4 | 47.0 ± 3.2 | 40.8 ± 2.2 | 40.7 ± 2.4 |

Data are presented as mean ± SE. *age-standardized rate (standardized to the population in 2005).

**Supplementary Table 12.** Adequate control of hypertension among hypertensive subjects in women, 1998‒2015

|  | 1998 | 2001 | 2005 | 2007-2009 | 2010-2012 | 2013-2015 |
| --- | --- | --- | --- | --- | --- | --- |
| Control | 6.6 ± 0.9 | 16.6 ± 1.6 | 35.5 ± 2.5 | 49.4 ± 1.3 | 48.2 ± 1.3 | 52.7 ± 1.2 |
| Control (standardized) | 6.6 ± 0.9 | 15.5 ± 1.5 | 32.7 ± 2.5 | 46.3 ± 1.4 | 43.3 ± 1.4 | 47.3 ± 1.3 |
| Age (years) |  |  |  |  |  |  |
| 30-39 | 1.7 ± 1.7 | 8.2 ± 5.2 | 6.7 ± 6.5 | 15.4 ± 5.6 | 17.8 ± 6.4 | 10.7 ± 5.4 |
| 40-49 | 7.5 ± 2.2 | 9.2 ± 2.8 | 26.0 ± 6.3 | 40.0 ± 3.5 | 31.7 ± 3.6 | 44.2 ± 4.1 |
| 50-59 | 7.2 ± 1.7 | 15.1 ± 3.2 | 37.8 ± 4.5 | 47.4 ± 2.7 | 44.7 ± 2.4 | 45.5 ± 2.5 |
| 60-69 | 8.2 ± 2.2 | 22.1 ± 3.3 | 39.0 ± 4.0 | 58.9 ± 2.3 | 51.7 ± 2.0 | 57.9 ± 2.1 |
| 70+ | 5.1 ± 1.6 | 17.9 ± 3.3 | 37.5 ± 4.3 | 50.6 ± 1.9 | 55.7 ± 1.9 | 58.2 ± 1.9 |
| Area of residence* |  |  |  |  |  |  |
| Urban area | 7.2 ± 1.2 | 15.0 ± 1.8 | 32.1 ± 2.6 | 46.4 ± 1.6 | 42.7 ± 1.6 | 46.8 ± 1.5 |
| Rural area | 5.2 ± 1.4 | 18.1 ± 2.8 | 30.6 ± 4.2 | 46.1 ± 2.5 | 45.1 ± 2.9 | 48.4 ± 2.8 |
| Income quartiles* |  |  |  |  |  |  |
| Highest | 4.5 ± 1.4 | 18.3 ± 3.1 | 34.9 ± 4.6 | 44.1 ± 2.7 | 43.2 ± 2.5 | 46.9 ± 2.4 |
| Upper middle | 5.6 ± 1.7 | 13.9 ± 2.8 | 29.2 ± 4.4 | 44.9 ± 2.4 | 42.6 ± 2.4 | 46.9 ± 2.6 |
| Lower middle | 6.9 ± 2.1 | 14.6 ± 3.2 | 35.2 ± 4.1 | 49.7 ± 3.0 | 46.0 ± 2.8 | 49.8 ± 2.7 |
| Lowest | 8.9 ± 2.1 | 13.8 ± 2.5 | 30.3 ± 4.3 | 46.6 ± 2.7 | 38.1 ± 2.0 | 44.7 ± 2.8 |
| Education levels* |  |  |  |  |  |  |
| Primary | 5.4 ± 1.0 | 14.3 ± 2.3 | 33.6 ± 4.2 | 41.8 ± 2.0 | 54.6 ± 3.9 | 57.1 ± 3.7 |
| Middle | 6.6 ± 2.8 | 27.8 ± 4.9 | 37.2 ± 5.6 | 48.0 ± 3.5 | 42.4 ± 2.9 | 49.4 ± 4.4 |
| High | 7.2 ± 2.7 | 15.4 ± 5.2 | 36.4 ± 5.2 | 54.4 ± 2.8 | 43.4 ± 2.9 | 47.4 ± 2.7 |
| University/college | 4.8 ± 3.7 | 14.6 ± 6.7 | 49.4 ± 9.6 | 74.1 ± 5.9 | 34.4 ± 5.1 | 45.2 ± 4.0 |

Data are presented as mean ± SE. *age-standardized rate

**Supplementary Table 13.** Adequate control of hypertension among subjects receiving antihypertensive medications, 1998‒2015

|  | 1998 | 2001 | 2005 | 2007-2009 | 2010-2012 | 2013-2015 |
| --- | --- | --- | --- | --- | --- | --- |
| Control | 23.8 ± 2.5 | 37.6 ± 2.6 | 54.9 ± 2.6 | 69.3 ± 1.2 | 69.1 ± 1.0 | 72.0 ± 1.0 |
| Control (standardized) | - | - | - | 69.2 ± 1.6 | 69.4 ± 1.4 | 71.9 ± 1.4 |
| Age (years) |  |  |  |  |  |  |
| 30-39 | - | - | - | 64.4 ± 8.6 | 68.5 ± 8.6 | 64.7 ± 9.3 |
| 40-49 | 34.2 ± 7.2 | 38.8 ± 7.0 | 53.9 ± 7.5 | 70.5 ± 3.4 | 70.4 ± 3.6 | 74.9 ± 3.5 |
| 50-59 | 26.9 ± 4.7 | 39.9 ± 5.4 | 59.8 ± 4.7 | 69.8 ± 2.3 | 70.3 ± 2.0 | 70.7 ± 2.2 |
| 60-69 | 21.3 ± 4.5 | 35.7 ± 4.0 | 53.4 ± 4.0 | 71.4 ± 1.7 | 68.8 ± 1.7 | 74.5 ± 1.6 |
| 70+ | 16.3 ± 3.8 | 36.7 ± 5.2 | 50.4 ± 4.3 | 66.4 ± 1.8 | 68.1 ± 1.5 | 70.4 ± 1.5 |
| Area of residence* |  |  |  |  |  |  |
| Urban area | 25.0 ± 3.2 | 36.6 ± 3.3 | 55.0 ± 3.2 | 69.5 ± 1.5 | 69.1 ± 1.2 | 72.4 ± 1.1 |
| Rural area | 20.7 ± 3.2 | 40.4 ± 4.1 | 54.8 ± 4.8 | 68.7 ± 1.8 | 69.1 ± 1.9 | 70.9 ± 2.1 |
| Income quartiles* |  |  |  |  |  |  |
| Highest | 27.7 ± 4.5 | 42.3 ± 5.7 | 53.1 ± 5.4 | 69.8 ± 2.2 | 68.2 ± 2.1 | 71.0 ± 1.9 |
| Upper middle | 23.8 ± 5.4 | 36.1 ± 5.5 | 58.6 ± 4.5 | 71.2 ± 2.0 | 70.4 ± 1.9 | 75.1 ± 1.8 |
| Lower middle | 25.2 ± 4.9 | 33.1 ± 5.3 | 50.7 ± 4.1 | 69.6 ± 2.1 | 70.4 ± 1.9 | 75.1 ± 1.8 |
| Lowest | 17.4 ± 3.8 | 38.7 ± 4.9 | 57.3 ± 4.8 | 66.0 ± 2.4 | 68.4 ± 1.8 | 73.1 ± 1.8 |
| Education levels* |  |  |  |  |  |  |
| Primary | 19.0 ± 2.6 | 36.5 ± 3.5 | 52.6 ± 3.3 | 67.1 ± 1.5 | 69.0 ± 1.4 | 70.4 ± 1.4 |
| Middle | 27.5 ± 6.6 | 45.9 ± 6.6 | 64.4 ± 5.8 | 69.1 ± 2.7 | 70.7 ± 2.5 | 74.1 ± 2.5 |
| High | 30.8 ± 6.1 | 37.5 ± 5.6 | 49.0 ± 4.8 | 73.5 ± 2.3 | 68.9 ± 2.2 | 75.9 ± 1.7 |
| University/college | 37.7 ± 9.9 | 28.4 ± 7.4 | 60.0 ± 7.7 | 71.7 ± 3.4 | 68.5 ± 3.2 | 70.9 ± 2.7 |

Data are presented as mean ± SE. *Rates were not age-standardized because of the small sample size in each age subgroups.

**Supplementary Table 14.** Adequate control of hypertension among subjects receiving antihypertensive medications in men, 1998‒2015

|  | 1998 | 2001 | 2005 | 2007-2009 | 2010-2012 | 2013-2015 |
| --- | --- | --- | --- | --- | --- | --- |
| Control | 23.5 ± 3.8 | 30.2 ± 4.1 | 51.1 ± 4.0 | 68.7 ± 1.9 | 71.8 ± 1.5 | 72.4 ± 1.4 |
| Control (standardized) | - | - | - | 68.2 ± 2.1 | 71.5 ± 1.8 | 71.1 ± 1.7 |
| Age (years) |  |  |  |  |  |  |
| 30-39 | - | - | - | 63.3 ± 10.7 | 72.1 ± 10.3 | 64.9 ± 10.3 |
| 40-49 | 28.2 ± 9.3 | 36.7 ± 10.4 | 43.9 ± 9.3 | 66.8 ± 5.0 | 69.1 ± 4.8 | 67.1 ± 4.8 |
| 50-59 | 27.1 ± 7.7 | 35.4 ± 8.3 | 57.7 ± 7.1 | 70.1 ± 3.1 | 71.6 ± 2.9 | 68.0 ± 3.3 |
| 60-69 | 17.5 ± 6.3 | 24.6 ± 5.9 | 53.5 ± 6.2 | 70.5 ± 2.6 | 71.8 ± 2.5 | 77.0 ± 2.3 |
| 70+ | 17.0 ± 7.4 | 28.5 ± 8.1 | 36.4 ± 7.1 | 66.4 ± 3.2 | 73.4 ± 2.4 | 75.3 ± 2.3 |
| Area of residence* |  |  |  |  |  |  |
| Urban area | 22.8 ± 4.4 | 30.0 ± 4.7 | 51.7 ± 4.8 | 68.8 ± 2.2 | 71.5 ± 1.7 | 73.6 ± 1.6 |
| Rural area | 26.3 ± 6.9 | 30.8 ± 8.3 | 48.6 ± 6.6 | 68.4 ± 3.2 | 72.8 ± 2.7 | 67.7 ± 3.2 |
| Income quartiles* |  |  |  |  |  |  |
| Highest | 27.8 ± 6.9 | 34.2 ± 7.9 | 49.8 ± 7.6 | 71.5 ± 3.3 | 69.6 ± 3.2 | 71.9 ± 2.9 |
| Upper middle | 19.9 ± 8.0 | 27.6 ± 7.2 | 52.4 ± 7.7 | 71.6 ± 3.3 | 69.9 ± 3.0 | 74.5 ± 2.9 |
| Lower middle | 24.9 ± 7.6 | 20.6 ± 7.3 | 50.4 ± 6.6 | 69.3 ± 3.5 | 77.6 ± 2.5 | 67.8 ± 3.1 |
| Lowest | 20.1 ± 7.1 | 34.8 ± 8.2 | 51.2 ± 2.4 | 63.4 ± 3.8 | 71.3 ± 2.9 | 75.3 ± 2.8 |
| Education levels* |  |  |  |  |  |  |
| Primary | 17.5 ± 5.2 | 27.3 ± 6.7 | 49.8 ± 7.1 | 66.7 ± 3.2 | 76.3 ± 2.8 | 70.5 ± 2.8 |
| Middle | 23.9 ± 10.7 | 28.3 ± 10.3 | 60.1 ± 8.4 | 64.4 ± 4.1 | 72.5 ± 3.6 | 77.9 ± 3.4 |
| High | 21.4 ± 6.7 | 36.6 ± 7.5 | 41.7 ± 6.0 | 70.1 ± 3.2 | 69.6 ± 2.8 | 75.4 ± 2.2 |
| University/college | 36.2 ± 9.7 | 26.4 ± 7.5 | 58.4 ± 8.4 | 72.6 ± 3.7 | 68.3 ± 3.5 | 69.2 ± 3.4 |

Data are presented as mean ± SE. *Rates were not age-standardized because of the small sample size in each age subgroups.

**Supplementary Table 15.** Adequate control of hypertension among subjects receiving antihypertensive medications in women, 1998‒2015

|  | 1998 | 2001 | 2005 | 2007-2009 | 2010-2012 | 2013-2015 |
| --- | --- | --- | --- | --- | --- | --- |
| Control | 24.0 ± 3.0 | 42.0 ± 3.6 | 57.8 ± 3.1 | 69.7 ± 1.3 | 67.0 ± 1.3 | 71.7 ± 1.3 |
| Control (standardized) | - | - | - | - | - | - |
| Age (years) |  |  |  |  |  |  |
| 30-39 | - | - | - | - | - | - |
| 40-49 | 39.9 ± 10.1 | 40.6 ± 10.6 | 71.3 ± 8.7 | 74.8 ± 4.3 | 72.2 ± 5.3 | 89.7 ± 3.3 |
| 50-59 | 26.8 ± 6.0 | 42.9 ± 6.9 | 61.8 ± 6.1 | 69.5 ± 3.0 | 68.8 ± 2.9 | 73.7 ± 2.8 |
| 60-69 | 22.8 ± 5.7 | 41.5 ± 5.2 | 53.4 ± 4.9 | 72.1 ± 2.3 | 66.3 ± 2.2 | 72.3 ± 2.1 |
| 70+ | 16.1 ± 4.5 | 40.5 ± 5.9 | 55.5 ± 5.1 | 66.4 ± 2.0 | 65.6 ± 1.9 | 67.8 ± 1.9 |
| Area of residence* |  |  |  |  |  |  |
| Urban area | 26.4 ± 4.1 | 40.5 ± 4.3 | 57.7 ± 3.7 | 70.0 ± 1.6 | 67.1 ± 1.5 | 71.3 ± 1.5 |
| Rural area | 18.7 ± 3.8 | 46.0 ± 6.1 | 58.0 ± 5.7 | 68.9 ± 2.0 | 66.7 ± 2.5 | 73.3 ± 2.4 |
| Income quartiles* |  |  |  |  |  |  |
| Highest | 27.7 ± 5.5 | 47.6 ± 6.8 | 55.6 ± 7.0 | 68.3 ± 2.7 | 67.1 ± 2.6 | 70.2 ± 2.5 |
| Upper middle | 25.6 ± 7.0 | 41.5 ± 6.9 | 62.4 ± 8.5 | 70.9 ± 2.4 | 70.8 ± 2.4 | 75.6 ± 2.2 |
| Lower middle | 25.4 ± 6.6 | 40.7 ± 7.2 | 50.9 ± 5.5 | 69.8 ± 2.5 | 63.9 ± 2.7 | 70.3 ± 2.5 |
| Lowest | 16.2 ± 4.4 | 40.2 ± 6.1 | 61.1 ± 5.5 | 68.1 ± 2.7 | 66.3 ± 2.5 | 71.2 ± 2.3 |
| Education levels* |  |  |  |  |  |  |
| Primary | 19.4 ± 3.1 | 39.3 ± 4.4 | 53.4 ± 3.5 | 67.3 ± 1.6 | 66.6 ± 1.6 | 70.3 ± 1.8 |
| Middle | 29.7 ± 8.9 | 52.7 ± 8.2 | 68.4 ± 7.2 | 74.4 ± 3.5 | 68.8 ± 3.5 | 70.8 ± 3.4 |
| High | 48.1 ± 9.7 | 38.8 ± 8.1 | 64.7 ± 8.1 | 78.3 ± 2.9 | 67.8 ± 3.7 | 76.8 ± 2.6 |
| University/college | 48.7 ± 23.9 | 34.7 ± 14.1 | 70.8 ± 13.1 | 67.2 ± 7.2 | 69.5 ± 7.0 | 76.9 ± 4.4 |

Data are presented as mean ± SE. *Rates were not age-standardized because of the small sample size in each age subgroups.

**Supplementary Figure 5.** Age-standardized hypertension control among hypertensive subjects (A) in total study subjects, and according to (B) the area of residence, (C) income quartiles, and (D) education levels

| 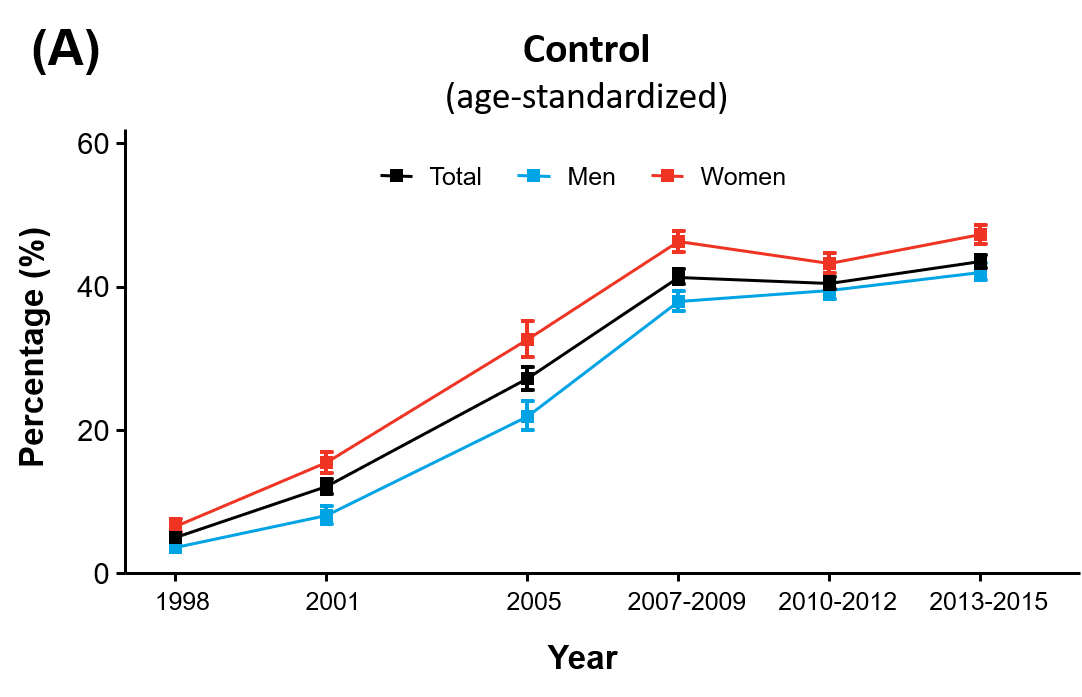 | 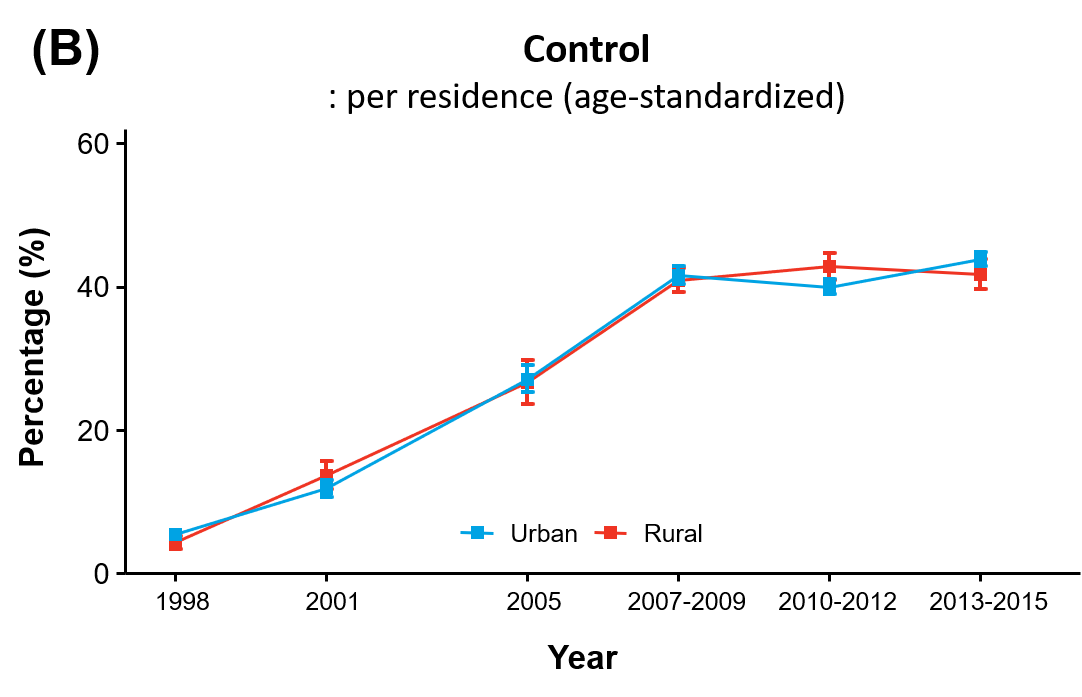 |
| --- | --- |
| 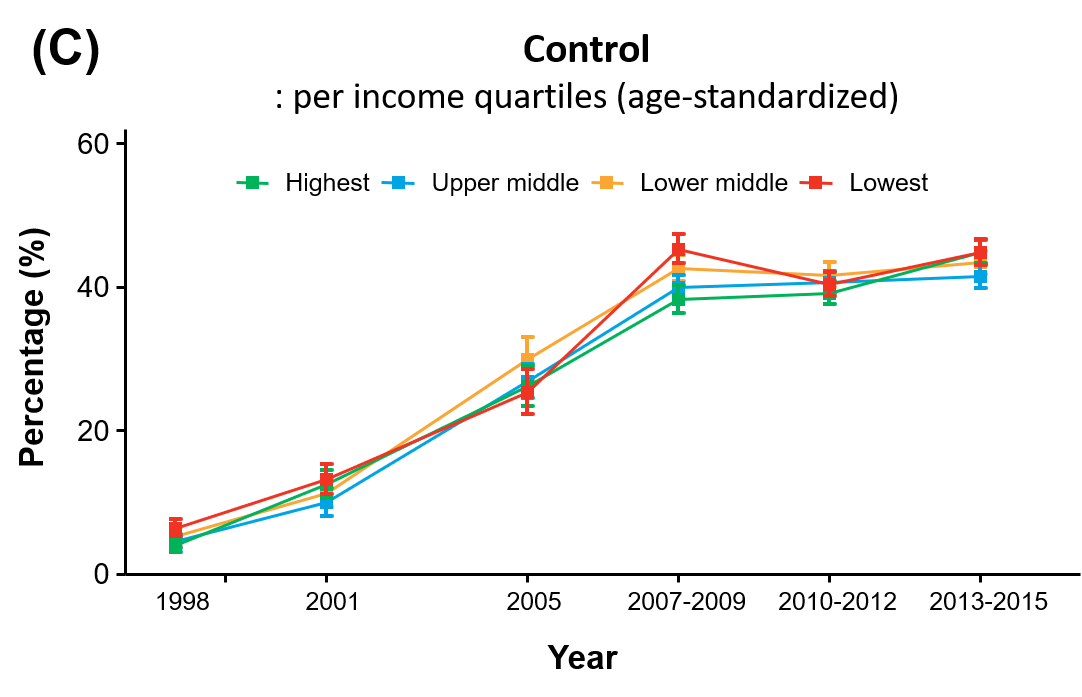 | 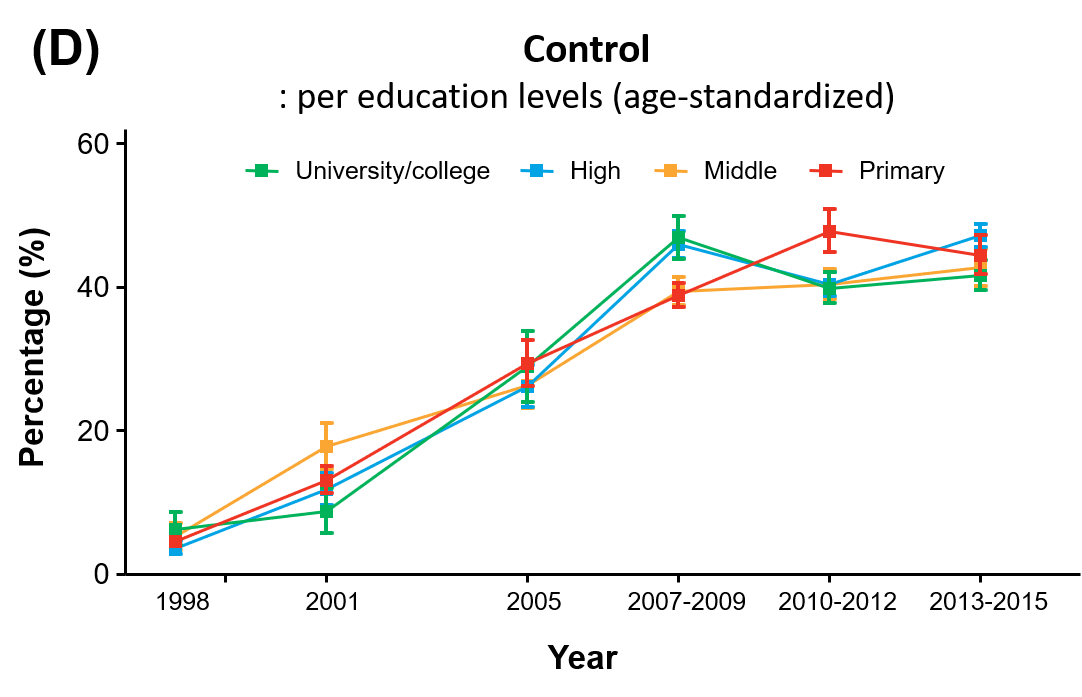 |

**Supplementary Figure 6.** Crude rate of hypertension control among subjects receiving antihypertensive medications according to (A) the area of residence, (B) income quartiles, and (C) education levels

| 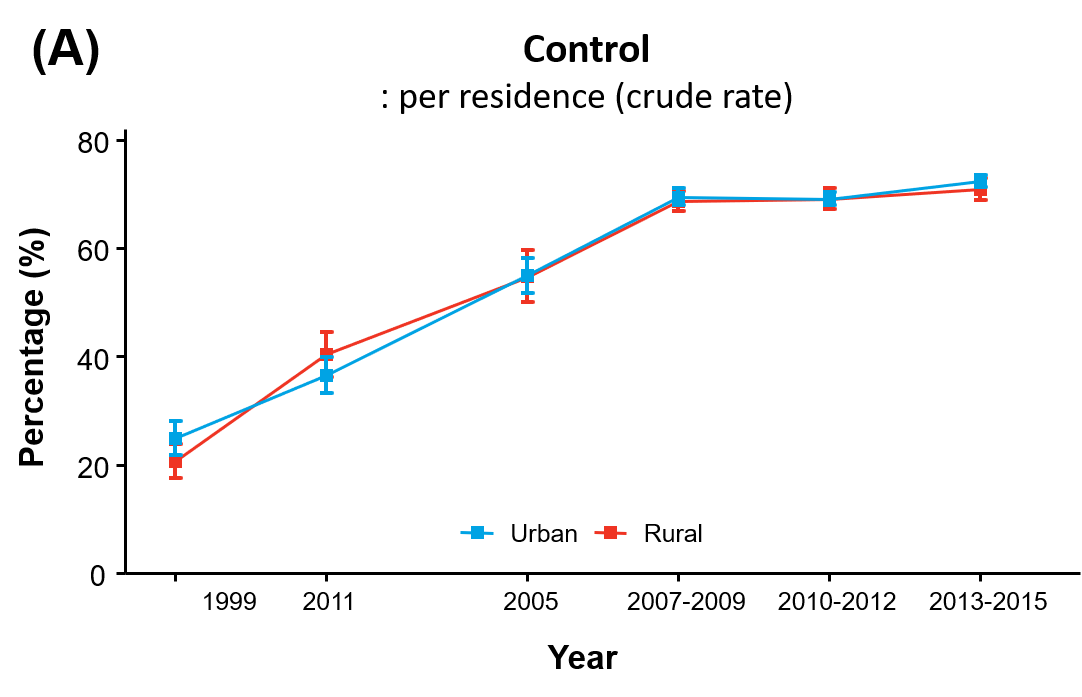 | 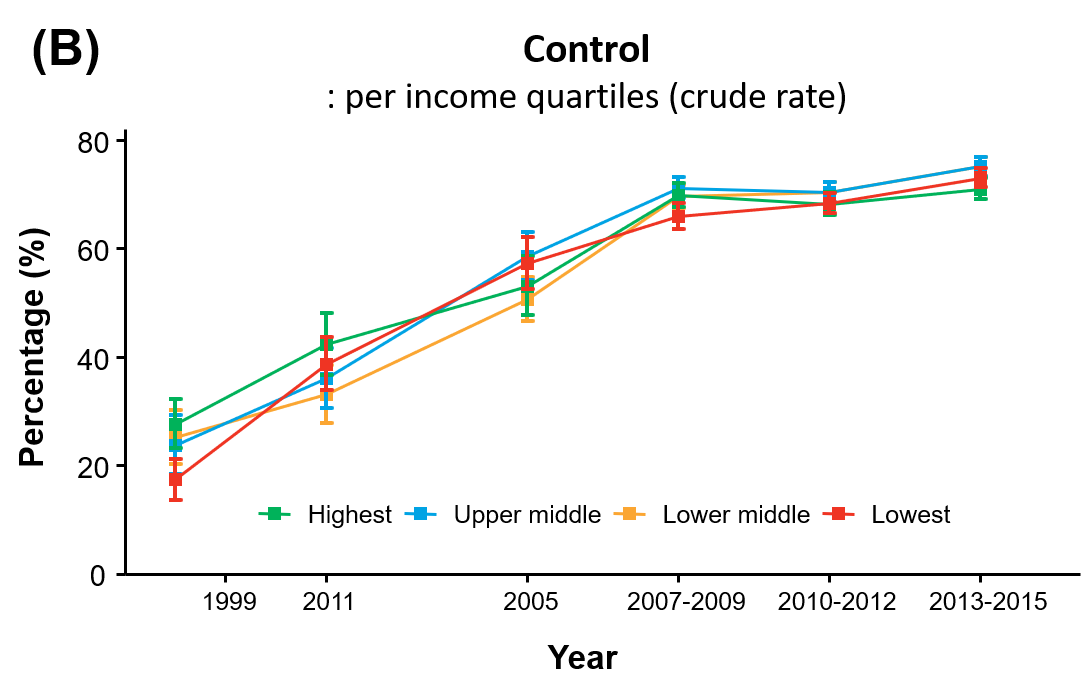 |
| --- | --- |
| 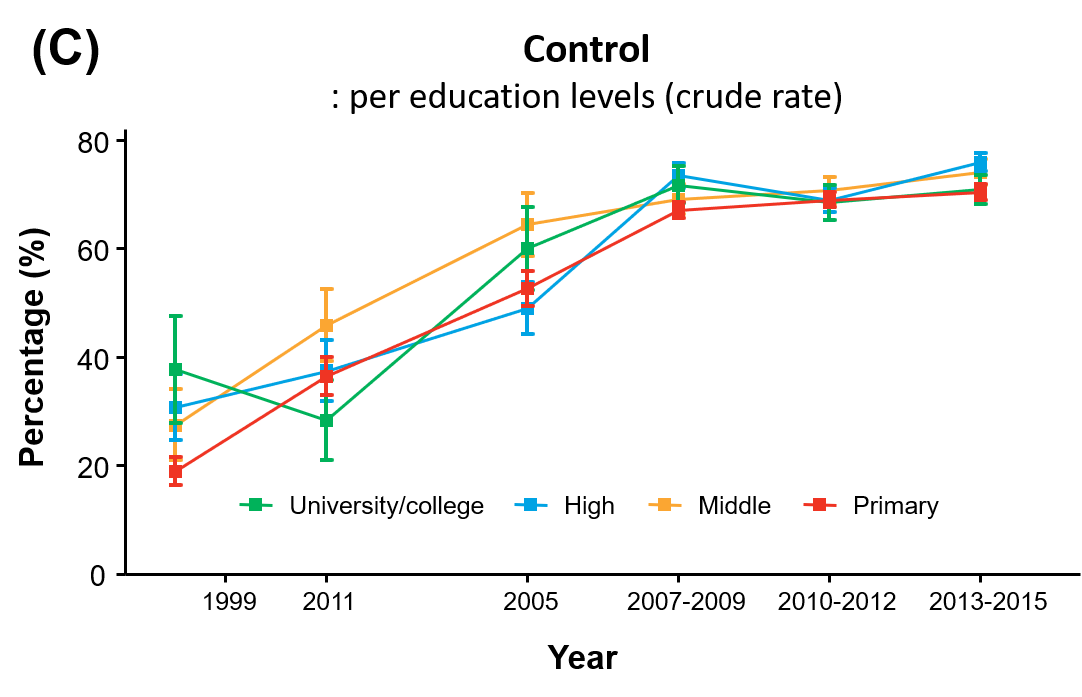 |  |

**Supplementary Table 16.** Comparison of the prevalence, awareness, treatment and control of hypertension across studies

|  | Korea | PURE | | | | | USA | Japan | | China | India |
| --- | --- | --- | --- | --- | --- | --- | --- | --- | --- | --- | --- |
|  |  | All | HI | UMIC | LMIC | LIC |  | Men | Women |  |  |
| Prevalence | 30.4% | 40.8% | 40.7% | 49.7% | 39.9% | 32.2% | 29.4% | 53.3% | 54.4% | 41.9% | 28.9% (rural)  42.2% (urban) |
| Awareness | 67.3% | 46.5% | 49.0% | 52.5% | 43.6% | 40.8% | 84.4% |  |  | 41.6% | 38.7% |
| Treatment | 63.6% | 40.6% | 46.7% | 48.3% | 36.9% | 31.7% | 74.7% |  |  | 34.4% | 32.3% |
| Control (1)* | 46.2% | 13.2% | 19.0% | 15.6% | 9.9% | 12.7% | 53.9% | 32.4% | 39.8% | 8.2% | 12.8% |
| Control (2)** | 72.0% | 32.5% | 40.7% | 31.7% | 12.7% | 40.2% | 73.0% |  |  | - | - |
| Study period | 2013‒2015 | 2003‒2009 | | | | | 2013‒2014 | 2010 | | 2005‒2009 | 2010‒2012 |
| Age criteria | ≥ 30 years | 35‒70 years | | | | | ≥ 18 years | ≥ 20 years | | 35‒70 years | 35‒64 years |

*Control (1), Controlled (among all participants with hypertension); **Control (2), Controlled (among participants treated for hypertension)

**Supplementary Figure 7.** Temporal trends in mean blood pressure according to age and sex


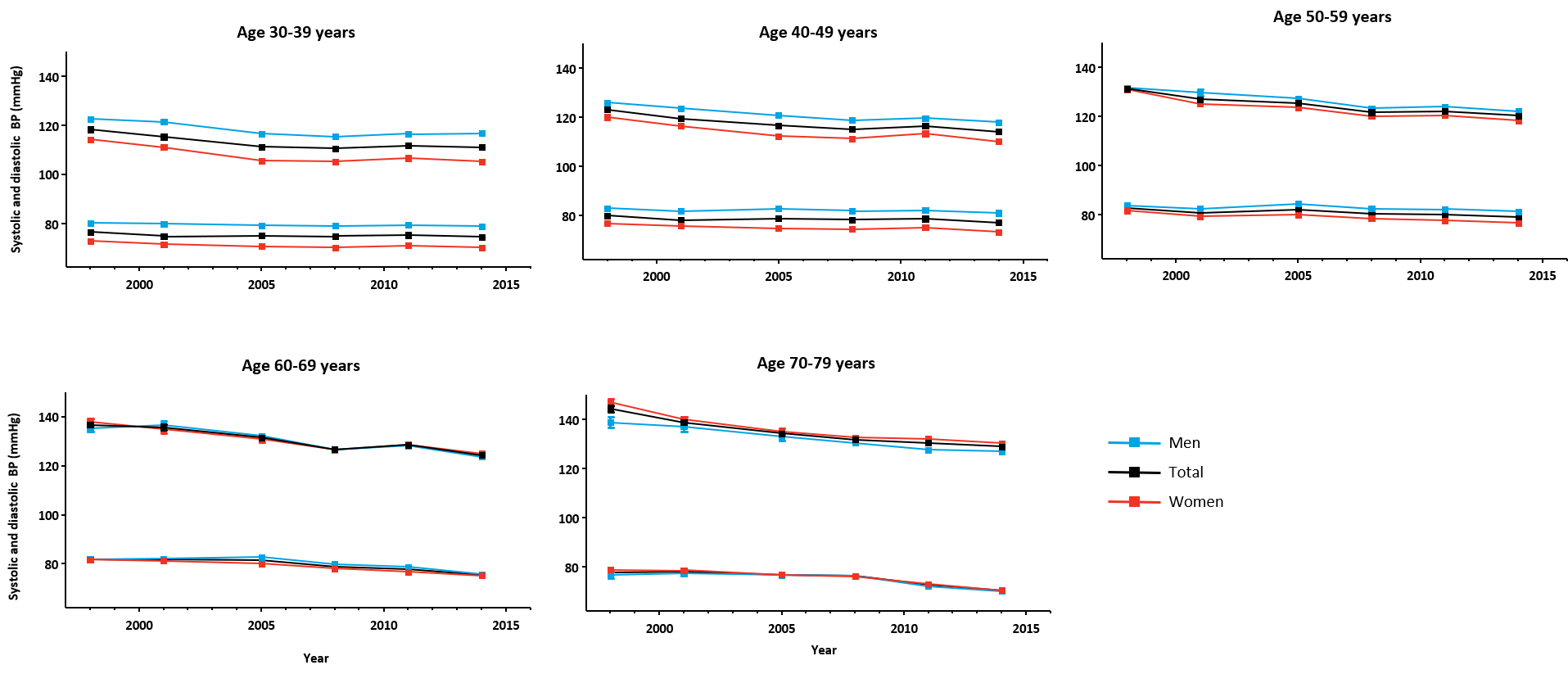

Supplement: Supplementary file 1 — Supplementary appendix [file 41598_2019_46965_MOESM1_ESM.docx]
